# Nitric Oxide‐Releasing Nanoscale Metal‐Organic Layer Overcomes Hypoxia and Reactive Oxygen Species Diffusion Barriers to Enhance Cancer Radiotherapy

**DOI:** 10.1002/advs.202413518

**Published:** 2025-01-01

**Authors:** Yuxuan Xiong, Jinhong Li, Xiaomin Jiang, Wenyao Zhen, Xin Ma, Wenbin Lin

**Affiliations:** ^1^ Department of Chemistry The University of Chicago Chicago IL 60637 USA; ^2^ Department of Radiation and Cellular Oncology and the Ludwig Center for Metastasis Research The University of Chicago Chicago IL 60637 USA

**Keywords:** hypoxia, metal‐organic layers, nitric oxide, radiotherapy, reactive oxygen species

## Abstract

Hafnium (Hf)‐based nanoscale metal‐organic layers (MOLs) enhance radiotherapeutic effects of tissue‐penetrating X‐rays via a unique radiotherapy‐radiodynamic therapy (RT‐RDT) process through efficient generation of hydroxy radical (RT) and singlet oxygen (RDT). However, their radiotherapeutic efficacy is limited by hypoxia in deep‐seated tumors and short half‐lives of reactive oxygen species (ROS). Herein the conjugation of a nitric oxide (NO) donor, S‐nitroso‐N‐acetyl‐DL‐penicillamine (SNAP), to the Hf_12_ secondary building units (SBUs) of Hf‐5,5′‐di‐p‐benzoatoporphyrin MOL is reported to afford SNAP/MOL for enhanced cancer radiotherapy. Under X‐ray irradiation, SNAP/MOL efficiently generates superoxide anion (O_2_ **^−.^**) and releases nitric oxide (NO) in a spatio‐temporally synchronized fashion. The released NO rapidly reacts with O_2_ **^−.^** to form long‐lived and highly cytotoxic peroxynitrite which diffuses freely to the cell nucleus and efficiently causes DNA double‐strand breaks. Meanwhile, the sustained release of NO from SNAP/MOL in the tumor microenvironment relieves tumor hypoxia to reduce radioresistance of tumor cells. Consequently, SNAP/MOL plus low‐dose X‐ray irradiation efficiently inhibits tumor growth and reduces metastasis in colorectal and triple‐negative breast cancer models.

## Graphical abstract

A nitric oxide‐releasing 2D metal‐organic layer is designed, SNAP/MOL, for overcoming tumor hypoxia and ROS diffusion barriers. Constructed from Hf_12_ SBUs and 5,5'‐di‐p‐benzoatoporphyrin bridging ligands, the MOL efficiently generates superoxide anion under X‐ray radiation. The spatio‐temporally synchronized generation of superoxide anion and release of nitric oxide facilitate the formation of long‐lived and highly cytotoxic peroxynitrite which efficiently diffuses into the nucleus to increase DNA damage. By alleviating tumor hypoxia, SNAP/MOL effectively enhances the local radiotherapy effect and suppresses distal pulmonary metastasis.

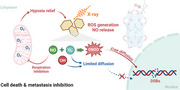

## 1 Introduction

Radiotherapy (RT) effectively controls localized tumors and oligometastases and improves quality of lives for patients with advanced tumors.^[^ ^1^ ^]^ When exposed to ionizing radiation such as X‐rays, γ‐rays, electrons, and protons, genomic DNAs of cancer cells can be damaged directly by secondary electrons and indirectly by reactive oxygen species (ROS), leading to DNA double‐stranded breaks (DSBs) and cell death.^[^ ^2^ ^]^ However, due to an intrinsically small difference in radiosensitivity between normal and cancer cells, high doses of radiation are needed to achieve favorable tumor control, which leads to unavoidable damage to healthy tissues and often debilitating side effects.^[^ ^2^, ^3^ ^]^

Nanoparticles containing high atomic number (Z) elements, such as Au and HfO_2_ nanoparticles and nanoscale metal‐organic frameworks (MOFs), have recently emerged as promising radiosensitizers to increase radiotoxicity to tumor cells by increasing energy deposition and ROS generation from X‐rays.^[^ ^4^ ^]^ These nanoradiosensitizers maintain efficient radiation damage to cancer cells while reducing X‐ray doses.^[^ ^5^ ^]^ In particular, we have shown enhanced energy deposition in nanoscale MOFs over solid nanoparticles due to the unique periodic arrangements of high‐Z secondary building units (SBUs) via Monte Carlo simulations^[^ ^6^ ^]^ and the ability of photosensitizer‐containing nanoscale MOFs in enhancing the radiotherapeutic effects via increased production of hydroxyl radicals (**·**OH) and the radiodynamic therapy (RDT) effect by generating singlet oxygen (^1^O_2_).^[^ ^7^ ^]^ Due to the unique RT‐RDT mechanism, MOF‐mediated radiotherapy elicits a more immunogenic tumor microenvironment (TME) for synergistic combination treatment with immune checkpoint blockade and other forms of immunotherapy.^[^ ^8^ ^]^ However, highly cytotoxic ROS generated by the RT‐RDT process have short half‐lives (≈10^−9^ seconds for **·**OH and ≈10^−6^ seconds for ^1^O_2_) and limited diffusion distances of less than 30 nm.^[^ ^9^ ^]^ As a result, the ROS have limited spatio‐temporal action ranges and only the ROS produced in the nuclei can efficiently induce DNA DSBs.^[^ ^10^ ^]^ Additionally, the inherent hypoxic nature of deep‐seated tumors also limits the generation of ROS from the RT‐RDT process, which further compromises the radiotherapeutic effects and leads to radioresistance of tumor cells.^[^ ^11^ ^]^ Therefore, innovative strategies are needed to overcome ROS diffusion barriers and tumor hypoxia to further enhance the therapeutic effects of MOF‐mediated radiotherapy.

As a multifunctional signaling and effector molecule, nitric oxide (NO) regulates a variety of physiological functions in humans.^[^ ^12^ ^]^ The intratumoral NO levels are positively correlated with tumor responses to anticancer therapies.^[^ ^13^ ^]^ Increasing evidence suggests that NO can alleviate tumor hypoxia by normalizing the vasculature to improving blood flow and oxygen supply and by modulating energy metabolism to reduce oxygen consumption and promote alternative metabolic pathways.^[^ ^14^ ^]^ A variety of organic (nitrate/nitrite and S‐nitrosothiols) and inorganic (metal nitroso complexes) compounds have been developed for NO delivery, but the low bioavailability and uncontrolled NO release have limited their therapeutic potency.^[^ ^15^ ^]^ Nanoparticles have recently been explored as an efficient means to deliver large quantity of NO to diseased tissues.^[^ ^16^ ^]^ Since NO contains an unpaired electron and is paramagnetic, it can readily react with O_2_ **^−.^** to form peroxynitrite (ONOO^−^). The reaction of O_2_ **^−.^** with NO has a five‐fold higher rate constant than the disproportionation reaction of O_2_ **^−.^** by superoxide dismutase (SOD) and ONOO^−^ formation is regarded as a diffusion‐controlled process.^[^ ^17^ ^]^ Thus, NO can effectively prevent the inactivation of O_2_^−.^ by SOD.^[^ ^18^ ^]^ Although both O_2_ **^−.^** and NO have limited chemical reactivity, the resultant ONOO^−^ is a potent oxidizing and nitrating agent with reactivity comparable to that of **·**OH, and can trigger cancer cell death via multiple mechanisms.^[^ ^19^ ^]^ More importantly, ONOO^−^ has a much longer half‐life of ≈1 s at pH 7.4 and 37 °C and a diffusion distance of up to 100 µm.^[^ ^20^ ^]^ This diffusion distance is sufficient for ONOO^−^ to move from the cytoplasm into the nucleus to react with DNAs (cell diameter ≈20 µm). Although NO is a relatively stable and highly diffusible radical, O_2_ **^−.^** is much shorter‐lived and its diffusion is severely limited due to its negative charge.^[^ ^21^ ^]^ Thus, spatio‐temporally synchronized formation of NO and O_2_ **^−.^** is critical for the efficient generation of ONOO^−^.

We recently developed synthetic strategies for two‐dimensional nanoscale metal‐organic layers (MOLs)^[^ ^22^ ^]^ and discovered that MOLs comprising high Z‐metal SBUs and photosensitizing ligands enhanced radiotherapy via the RT‐RDT process.^[^ ^7c^ ^]^ Unlike nanoscale MOFs, the monolayered structure of MOLs allows complete access to the SBUs and efficient loading of carboxy‐containing drugs and prodrugs via capping ligand exchange.^[^ ^23^ ^]^ We hypothesize that high‐Z MOLs could be loaded with NO‐releasing prodrugs to simultaneously overcome hypoxia and facilitate the generation of ONOO^−^ via spatio‐temporally synchronized formation of NO and O_2_ **^−.^** for enhanced radiotherapeutic efficacy.^[^ ^24^ ^]^

Herein we report the conjugation of a NO donor, S‐Nitroso‐N‐acetyl‐DL‐penicillamine (SNAP), to the Hf_12_ SBUs of Hf_12_‐5,5′‐di‐p‐benzoatoporphyrin (Hf‐DBP) nanoscale MOL for effective cancer radiotherapy. Under X‐ray irradiation, SNAP/MOL efficiently generates O_2_ **^−.^** and releases NO in a spatio‐temporally synchronized fashion.^[^ ^25^ ^]^ The released NO readily reacts with O_2_ **^−.^** to form long‐lived, highly cytotoxic ONOO^−^ which diffuses freely to the cell nucleus and efficiently causes DNA double‐strand breaks. Meanwhile, the sustained release of NO from SNAP/MOL in the TME relieves tumor hypoxia to reduce radioresistance of tumor cells. Consequently, SNAP/MOL plus low‐dose X‐ray irradiation efficiently inhibits tumor growth and reduces metastasis in CT‐26 colorectal and 4T1 triple‐negative breast cancer models.

## 2 Results and Discussion

### 2.1 Preparation and Characterization of SNAP/MOL

**Figure 1:**
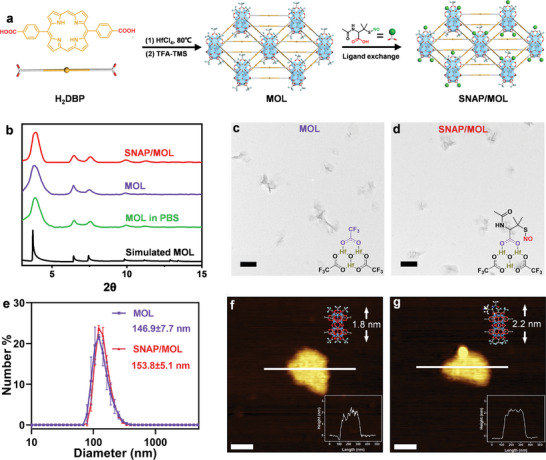
Characterization of SNAP/MOL. a) Synthetic scheme of SNAP/MOL. b) PXRD patterns of MOL and SNAP/MOL. TEM images of c) MOL and d) SNAP/MOL. Scale bar, 200 nm. e) Number‐average sizes of MOL and SNAP/MOL by DLS. The data are shown as mean±SD, *n* = 3. AFM topographic images and height profiles (insets) of f) MOL and g) SNAP/MOL. Scale bar, 100 nm.

Hf‐DBP MOL was synthesized via a solvothermal reaction of HfCl_4_ and H_2_DBP in N, N‐dimethylformamide (DMF) at 80 °C using propionic acid (PA) as modulator (**Figure** **1**a).^[^ ^24^ ^]^ Powder X‐ray diffraction (PXRD) studies showed that Hf‐DBP was built from Hf_12_(µ_3_‐O)_8_(µ_3_‐OH)_8_(µ_2_‐OH)_6_ SBUs and DBP bridging ligands to form a 2D network with a kagome double (kgd) topology (Figure 1b). Hf‐DBP was treated with trimethylsilyl trifluoroacetate to afford MOL with trifluoroacetate capping groups replacing the PA capping groups in Hf‐DBP, as confirmed by the apperance of the TFA peak in the ^19^F‐NMR spectrum (Figure S1, Supporting Information).^[^ ^26^ ^]^ MOL was then treated with SNAP at room temperature to afford SNAP/MOL with an empirical formula of Hf_12_(µ_3_‐O)_8_(µ_3_‐OH)_8_(µ_2_‐OH)_6_(DBP)_6_(µ_2_‐TFA)_2.4_(µ_2_‐SNAP)_3.6_ as established by ^1^H NMR spectroscopy (Figure S2, Supporting Information).

Transmission electron microscopy (TEM) imaging indicated that MOL and SNAP/MOL adopted ultrathin nanoplate morphologies of approximately 170 and 180 nm in diameters (Figure 1c,d). Dynamic light‐scattering (DLS) studies gave hydrodynamic sizes of 146.9±7.7 and 153.8±5.1 nm for MOL and SNAP/MOL, respectively (Figure 1e). Atomic force microscopy (AFM) imaging demonstrated the monolayer structure of MOL and SNAP/MOL, with thicknesses of 1.8 nm and 2.2 nm, respectively. The slight height increase in SNAP/MOL is consistent with the larger size of SNAP over TFA (Figure 1f,g). Meanwhile, the ζ potential of MOL and SNAP/MOL were −9.6 and −3.5 mV, respectively. The changes in surface potentials also supported the modification of SNAP (Figure S5, Supporting Information). PXRD studies further showed that SNAP loading did not affect the crystalline structure of MOL (Figure 1b), and SNAP/MOL retained crystallinity after incubation in different physiologically relevant media (Figure S6, Supporting Information). Thus, SNAP/MOL has excellent stability for biological applications.

### 2.2 X‐ray Triggered ROS Generation and NO Release

**Figure 2:**
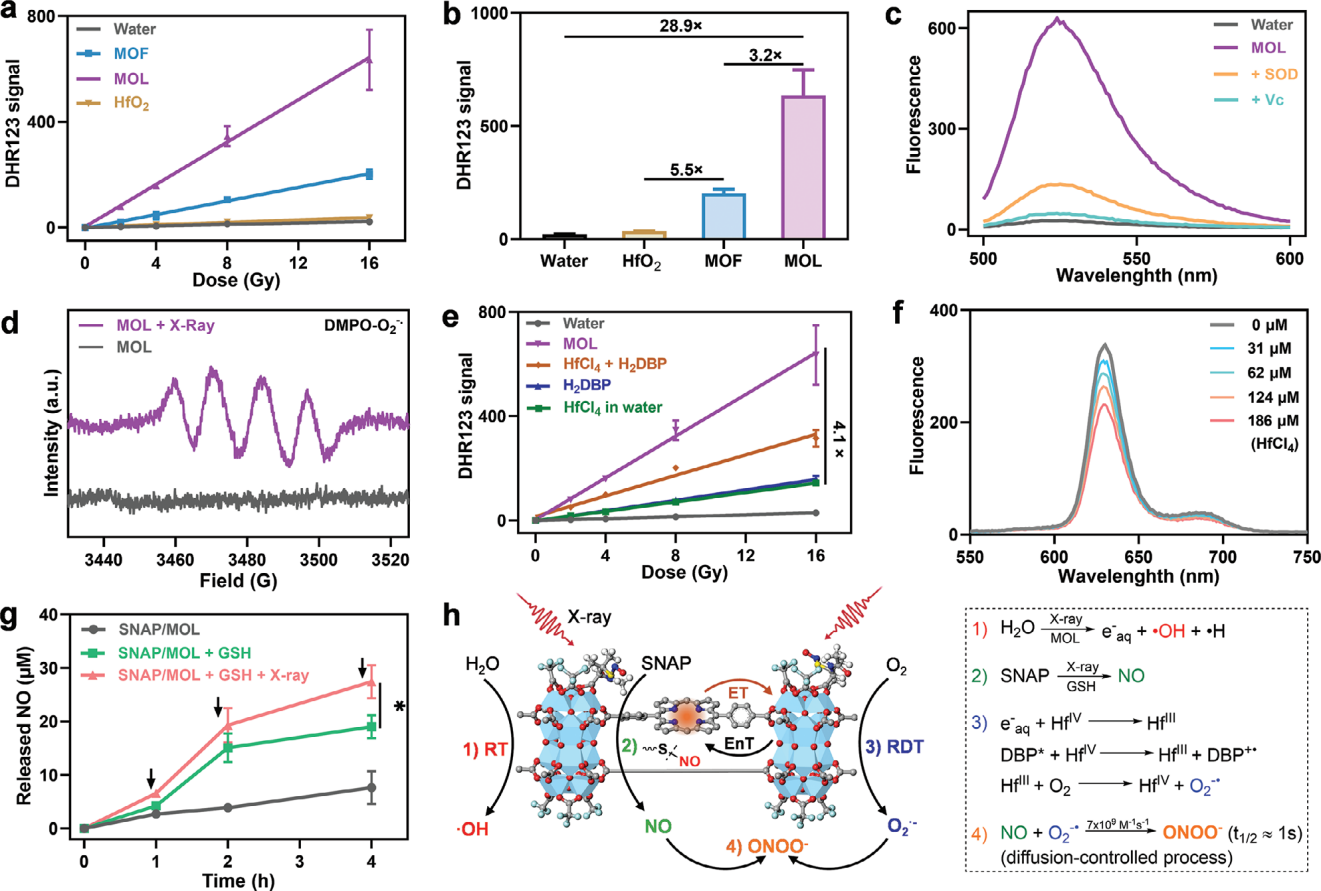
ROS generation and NO release under X‐ray irradiation. a) DHR123 assay showing O_2_^−.^ generation by MOL, MOF, and HfO_2_ at different doses of X‐ray irradiation. b) of DHR123 fluorescence intensities for MOL, MOF, and HfO_2_ at 528 nm after 16 Gy X‐ray irradiation. c) DHR123 fluorescence spectra of MOL plus X‐ray irradiation with the addition of SOD (50 µg mL^−1^) and Vc (50 µM). d) EPR signals of DMPO‐O_2_^−.^ adduct formed by MOL plus X‐ray irradiation. e) O_2_^−.^ generation by MOL, HfCl_4_, and H_2_DBP in aqueous solution at different doses of X‐ray irradiation. f) Emission spectra of 20 µM H_2_DBP with addition of different amounts of HfCl_4_. Ex: 405 nm. g) The release of NO from SNAP/MOL under different conditions. The black arrow represents 8 Gy of X‐ray irradiation. h) Schematic illustration of X‐ray induced ROS generation by and NO release from SNAP/MOL. All data are shown as mean±SD, *n* = 3. Statistical significance was calculated via unpaired two‐tailed student's t test. *p<0.05.

Radiolysis of water by ionizing radiation such as X‐rays produces several highly reactive species, namely hydroxyl radicals (·OH), hydrated electrons (e^−^_aq_), and hydrogen radicals (H·).^[^ ^27^ ^]^ Among these, ·OH is the predominant reactive species for causing DNA damage and cell death.^[^ ^28^ ^]^ We used aminophenyl fluorescein (APF) probe to detect ·OH production by MOL under X‐ray irradiation. A dispersion of MOL in PBS at a Hf concentration of 40 µM showed 1.3‐fold higher ·OH yield than pure water at 8 Gy of X‐ray (Figure S9, Supporting Information), likely due to the strong absorption of X‐rays by Hf_12_ SBUs.^[^ ^29^ ^]^ In the presence of oxygen, e^−^_aq_ and H· produced by ionizing radiation can be converted to O_2_^−.^, a potent ROS with 6 orders of magnitude longer lifetime than ·OH.^[^ ^27a^ ^]^ We measured O_2_^−.^ production with dihydrorhodamine 123 (DHR123) under X‐ray irradiation.^[^ ^30^ ^]^ Surprisingly, we found that MOL enhanced O_2_^−.^ production with linear dependence on X‐ray doses and Hf concentrations (**Figure** **2**a; Figure S10, Supporting Information) and MOL in water (40 µM Hf) produced 28.9‐fold more O_2_^−.^ than pure water at an X‐ray dose of 16 Gy (Figure 2b). Furthermore, the addition of SOD or ascorbic acid (Vc) to the MOL aqueous dispersions markedly decreased the fluorescence of DHR123 to the background level (Figure 2c; Figure S11, Supporting Information), supporting the enhanced production of O_2_^−.^ by MOL.^[^ ^25a^ ^]^ Electron paramagnetic resonance (EPR) signals characteristic of the DMPO‐O_2_^−.^ adduct were observed for a methanol dispersion of the MOL under X‐ray irradiation, further supporting the generation of O_2_^−.^ (Figure 2d).^[^ ^31^ ^]^

The mechanism of MOL‐mediated O_2_^−.^ generation was next investigated. Under X‐ray irradiation, HfCl_4_ or H_2_DBP produced 4.1‐fold less O_2_^−.^ than MOL while a mixture of HfCl_4_ or H_2_DBP produced a 2.0‐fold less O_2_^−.^ than MOL (Figure 2e). This result suggests that Hf^IV^ centers and DBP ligands synergistically promote O_2_^−.^ production. H_2_DBP luminescence was quenched by HfCl_4_ with a Stern‐Völmer constant of 2.34 mM^−1^, suggesting an electron transfer (ET) process from H_2_DBP excited states to Hf^IV^ centers (Figure 2f).^[^ ^32^ ^]^ Consistent with this, the fluorescence of DBP ligands was significantly quenched in MOL (Figure S12, Supporting Information).

The standard electrode potential of Hf^III^/Hf^IV^ couples in aqueous solution is −1.70 V (versus SHE).^[^ ^33^ ^]^ Radiation‐produced e^−^_aq_ has high reactivity with a reduction potential at −2.87 V (vs SHE).^[^ ^34^ ^]^ Thus, radiation‐generated e^−^_aq_ can reduce Hf^IV^ centers to generate Hf^III^ centers. Upon treatment with O_2_, cryogenic EPR spectrum of oxidized Hf^III^H‐DBP showed two sets of EPR signals for Hf‐O_2_^·^. species and Hf^III^ centers (Figure S13, Supporting Information), indicating Hf^III^ centers formed by ET from the DBP excited state and by e^−^_aq_ reduction can react with O_2_ to produce O_2_^−[^ ^33^ ^]^

We also examined O_2_^−.^ generation by three‐dimensional (3D) Hf‐MOF with the same Hf_12_ SBUs and DBP bridging ligands to understand the influence of the monolayer structure of MOL on O_2_^−.^ generation. As shown in Figure 2a,b, MOL generated 3.2‐fold more O_2_^−.^ than MOF, likely due to more facile oxygen diffusion and contact with the Hf^III^ centers in the MOL.^[^ ^24^ ^]^ Furthermore, a Hf‐MOF based on Hf_12_ SBUs and non‐photosensitizing amino‐quaterphenyldicarboxylic acid (QP) ligands also produced 75.2% of O_2_^−.^ as Hf‐DBP MOF with photosensitizing DBP ligands (Figure S14, Supporting Information), which supports the important role of e^−^_aq_ reduction of Hf^IV^ centers to produce O_2_^−.^

We also used HfO_2_ nanoparticles of 61–80 nm in size as a control. HfO_2_ nanoparticles are currently examined as a radioenhancer in clinical trials.^[^ ^35^ ^]^ At 16 Gy irradiation, solid HfO_2_ nanoparticles produced 5.5‐ and 17.6‐fold less O_2_^−.^ than MOF and MOL, respectively, which can be attributed to the inability for the bulk Hf centers in nonporous HfO_2_ particles to transfer electrons to O_2_.^[^ ^5c^ ^]^ Taken together, 2D MOL efficiently generates ROS, particularly ·OH and O_2_^−.^, under X‐ray irradiation. This finding augments the previous observation of ·OH and ^1^O_2_ generation from designer MOFs under X‐ray irradiation and provides a strong foundation for MOL‐mediated RT‐RDT.^[^ ^7a^ ^]^

The release profile of NO from SNAP/MOL was examined by the Griess detection method.^[^ ^36^ ^]^ First, the addition of glutathione (GSH) and the acidic condition enhanced NO release from SNAP/MOL (Figure S15b, Supporting Information), suggesting the ability to trigger NO release from SNAP/MOL in the TME.^[^ ^37^ ^]^ X‐ray irradiation at 8 Gy/fraction for three fractions further increased NO release by 1.44‐folds over GSH (Figure 2g), likely due to the cleavage of weak S‐N bonds in SNAP by high‐energy X‐ray irradiation.^[^ ^38^ ^]^ Moreover, the NO release was X‐ray dose‐dependent, which further supports X‐ray triggered NO release (Figure S15c, Supporting Information). Taken together, X‐ray not only generates O_2_^−.^ but also trigger the release of NO from SNAP/MOL (Figure 2h).

### 2.3 Intracellular ROS/RNS Generation and Radiosensitization of SNAP/MOL

**Figure 3:**
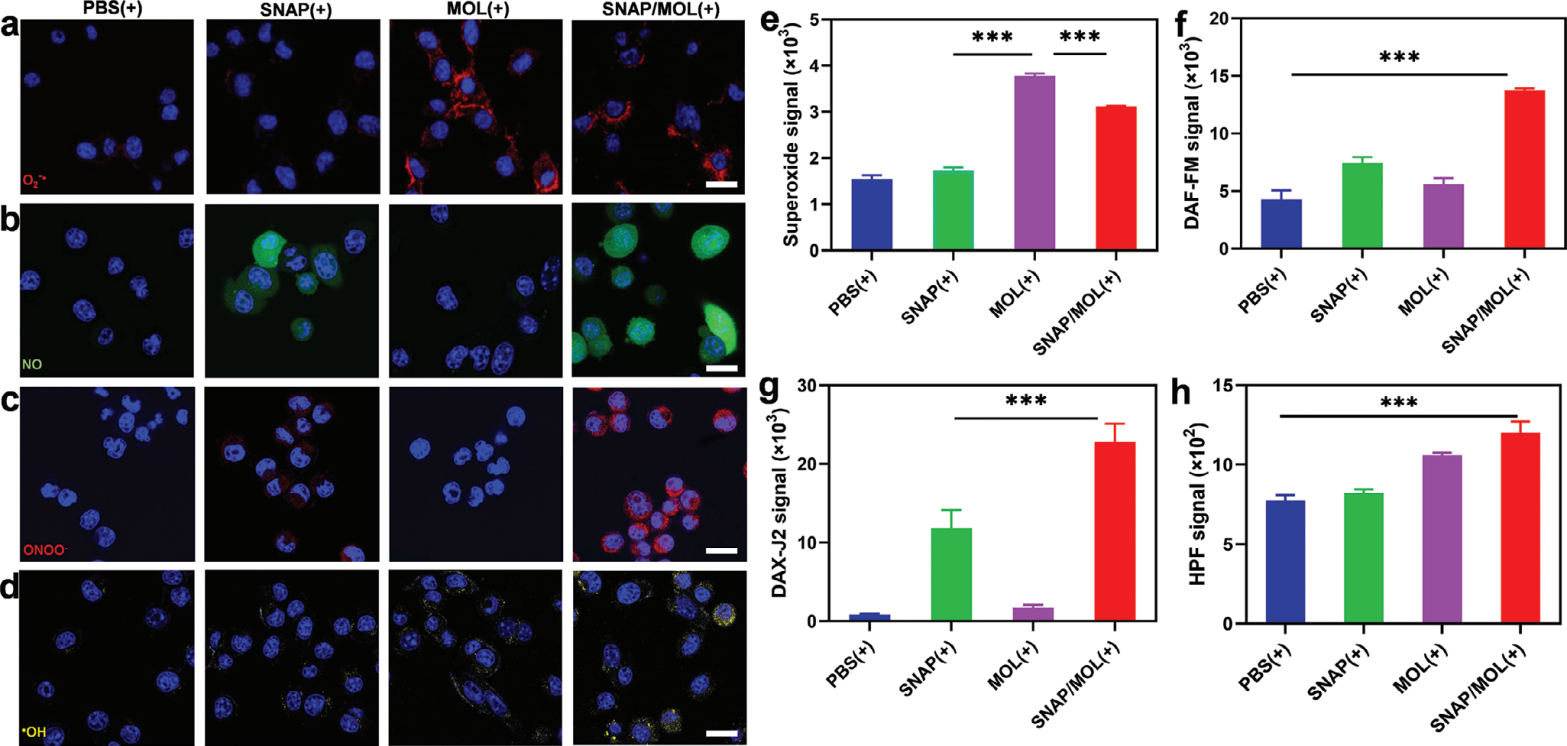
Intracellular ROS and reactive nitrogen species (RNS) generation. CLSM images of a) O_2_^−.^, b) NO, c) ONOO^−^ and d) ·OH staining after different treatments. The nuclei were visualized with Hoechst 33 342 (blue). e–h) Flow Cytometry analysis of (e) O_2_^−.^, (f) NO, (g) ONOO^−^ and (h) ·OH signals after different treatments. The scale bars are 50 µm in a–d. All data are shown as mean±SD, *n* = 3. The scale bars are 50 µm. Statistical significance was calculated via unpaired two‐tailed student's t test. ***p<0.001.

The ROS generation by and NO release from SNAP/MOL under X‐ray irradiation [denoted SNAP/MOL(+)] in 4T1 cells were studied by confocal laser scanning microscopy (CLSM) and flow cytometry. Intracellualr O_2_^−.^ and NO were probed by superoxide assay kit and 4‐amino‐5‐methylamino‐2′,7′‐difluorofluorescein diacetate (DAF‐FM DA), respectively. When stained with superoxide assay kit, 4T1 cells treated with MOL(+) and SNAP/MOL(+) showed bright red fluorescence due to O_2_^−.^ generation (**Figure** **3**a,e; Figure S17, Supporting Information). The red fluorescence was efficiently quenched by Vc, which further supported the production of O_2_^−.^ by MOL(+) and SNAP/MOL(+) (Figure S18, Supporting Information). Notably, SNAP/MOL(+) showed weaker O_2_^−.^ signals than MOL(+), suggesting partial O_2_^−.^ consumption by the NO released from SNAP/MOL.^[^ ^19a^ ^]^

DAF‐FM DA staining showed that SNAP/MOL(+) efficiently released NO, with 1.9‐fold higher DAF‐FM fluorescence than SNAP(+) (Figure 3b,f; Figure S20, Supporting Information). In addition to its intrinsic regulatory functions, NO can react with O_2_^−.^ to generate highly cytotoxic ONOO^−^.^[^ ^39^ ^]^ However, as the half‐life and intracellular diffusion distance of O_2_^−.^ are short, spatio‐temporally synchronized production of NO and O_2_^−.^ is critical for the efficient formation of ONOO^−^.^[^ ^40^ ^]^ Anchored on Hf_12_ SBUs of SNAP/MOL, the released NO and the generated O_2_^−.^ are in close proximity of <2 nm (Figure S8, Supporting Information), which is expected to facilitate the formation of ONOO^−^. We used DAX‐J2 PON Green probe to detect ONOO^−^ in 4T1 cells after different treatments.^[^ ^41^ ^]^ SNAP/MOL(+) showed 12.9‐ and 1.7‐fold higher ONOO^−^ generation than MOL(+) and SNAP(+), respectively (Figure 3c,g). Interestingly, SNAP/MOL(+) generated 1.4‐fold higher ONOO^−^ than a simple combination treatment of SNAP + MOL(+), supporting the important role of NO and O_2_^−.^ proximity on ONOO^−^ generation (Figure S21, Supporting Information).

Last, we used the hydroxyphenyl fluorescein (HPF) probe to examine intracellular ·OH generation.^[^ ^42^ ^]^ MOL(+) and SNAP/MOL(+) produced significantly more ·OH than PBS(+), supporting the radiosensitizing effects of MOL (Figure 3d,h; Figure S19, Supporting Information).

**Figure 4:**
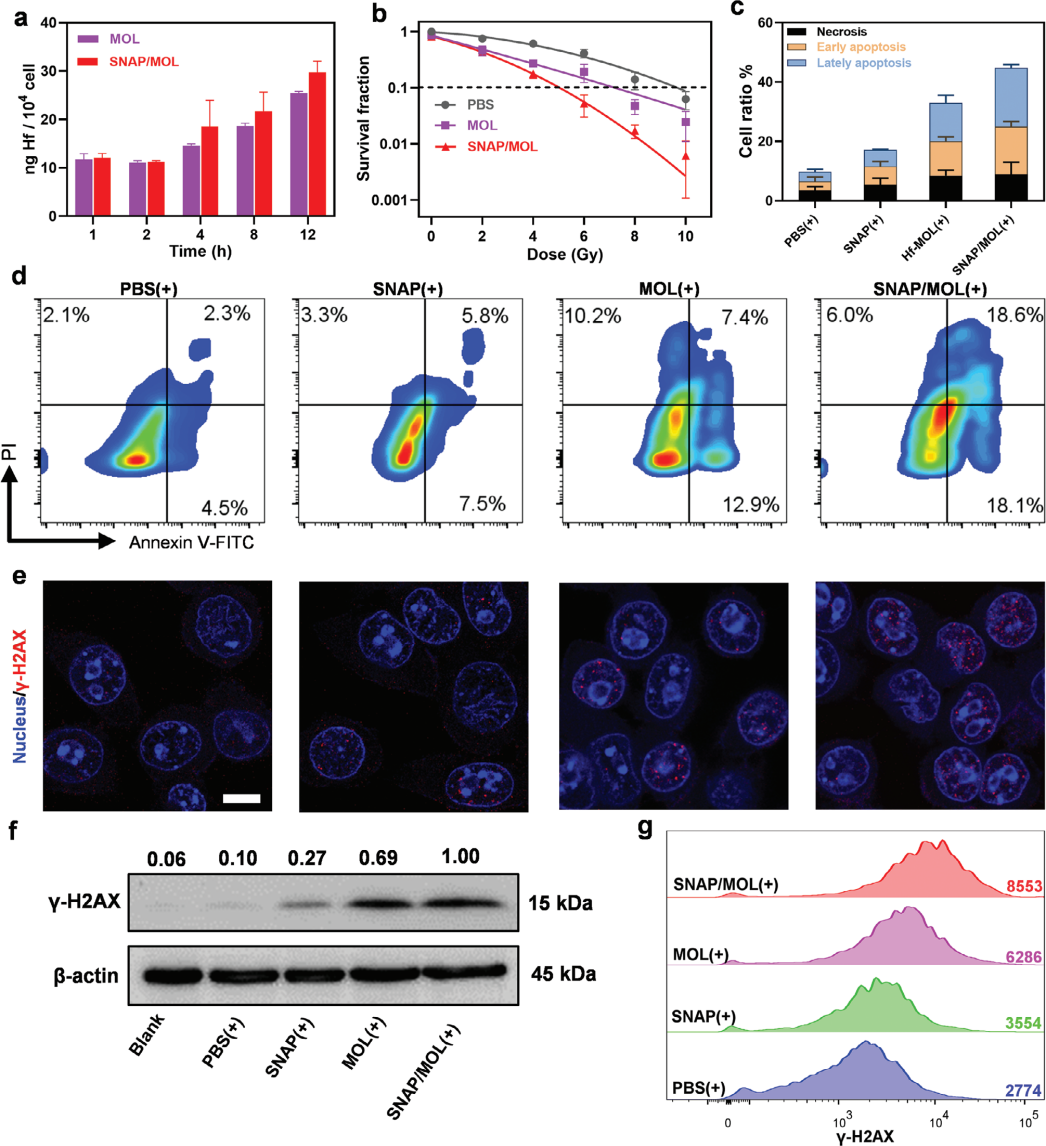
In vitro anticancer effect of SNAP/MOL under X‐ray irradiation. a) Cellular uptake of MOL and SNAP/MOL determined by ICP‐MS. b) Clonogenic assays to evaluate radioenhancement of MOL and SNAP/MOL in 4T1 cells. *n* = 3. c) Quantitation of cell apoptosis and necrosis after different treatments. *n* = 3. d) Representative flow cytometry results showing cell apoptosis and necrosis co‐stained with Annexin‐V and PI. e) CLSM images, f) western blot results, and g) flow cytometry analysis of γ‐H2AX signals after different treatments. All data are shown as mean±SD.

As high intracellular ROS and reactive nitrogen species (RNS) levels can cause cell death, we investigated the in vitro antitumor effects of SNAP/MOL(+) in 4T1 cells.^[^ ^43^ ^]^ ICP‐MS and flow cytometry results showed that MOL and SNAP/MOL were efficiently uptaken by 4T1 cells in a time‐dependent manner (**Figures** **4**a and S23). The radiosensitization of SNAP/MOL were next assessed by clonogenic assay, apoptotic test and DNA double‐strand break (DSB) quantifications in 4T1 cells. As a gold standard for assessing radiotherapy efficacy, the 10% survival fraction (DMR_10%_) dose modification ratio calculated from the clonogenic assay was used to evaluate the radiosensitizing effects.^[^ ^6^ ^]^ SNAP/MOL showed a DMR_10%_ value of 1.98, much higher than that of MOL (1.37), likely attributable to the radio‐potentiating effect of SNAP (Figure 4b; Figure S24, Supporting Information). SNAP/MOL(+) gave a sum of apoptotic and necrotic cells of 44.8%, significantly higher than those of MOL (32.3%), SNAP (17.2%) and PBS (9.8%) (Figure 4c,d).

As DSBs are the most lethal DNA lesions induced by ionizing radiation,^[^ ^44^ ^]^ we used γ‐H2AX assay to probe lethal DNA damages after different treatments. CLSM imaging showed that MOL(+) and SNAP/MOL(+) induced significantly higher γ‐H2AX fluorescence than SNAP(+) (Figure 4e). Analysis of fluorescence signals showed that SNAP/MOL(+) produced 1.6 times more DNA damage foci than MOL(+) (Figure S25, Supporting Information). We then used western blot analysis to quantify γ‐H2AX expressions after different treatments. MOL(+) upregulated γ‐H2AX protein by 6.9‐folds over PBS(+), indicating a strong radiosensitizing effect of MOL. SNAP/MOL(+) further increased γ‐H2AX protein expression by 1.4‐folds over MOL(+) (Figure 4f). A similar trend was observed in flow cytometric analysis (Figure 4g). These results indicate strong synergistic radiosensitizing effects between SNAP and MOL under X‐ray irradiation.

### 2.4 SNAP/MOL Overcomes ROS Diffusion Barriers and Tumor Hypoxia

We next examined the underlying mechanism for the enhanced DNA damage by SNAP/MOL(+). The ROS generated by RT‐RDT, ·OH, O_2_^−.^, and ^1^O_2_, have short lifetimes of 10^−9^‐10^−3^ s and diffusion distances of <30 nm in cellular environments.^[^ ^45^ ^]^ The ROS generated in the cytoplasm cannot reach the nucleus to damage DNA, thus compromising the radiotherapeutic effects of MOL(+). Thus, directly generating ROS in the nucleus and prolonging the lifetime of ROS are two strategies to enhance ROS‐mediated DNA damage.^[^ ^46^ ^]^ As CLSM images showed that SNAP/MOL predominantly distributed in the cytoplasm after cell entry (Figure S26, Supporting Information), the enhanced DNA damage by SNAP/MOL(+) likely resulted from increased ROS diffusion distances.

**Figure 5:**
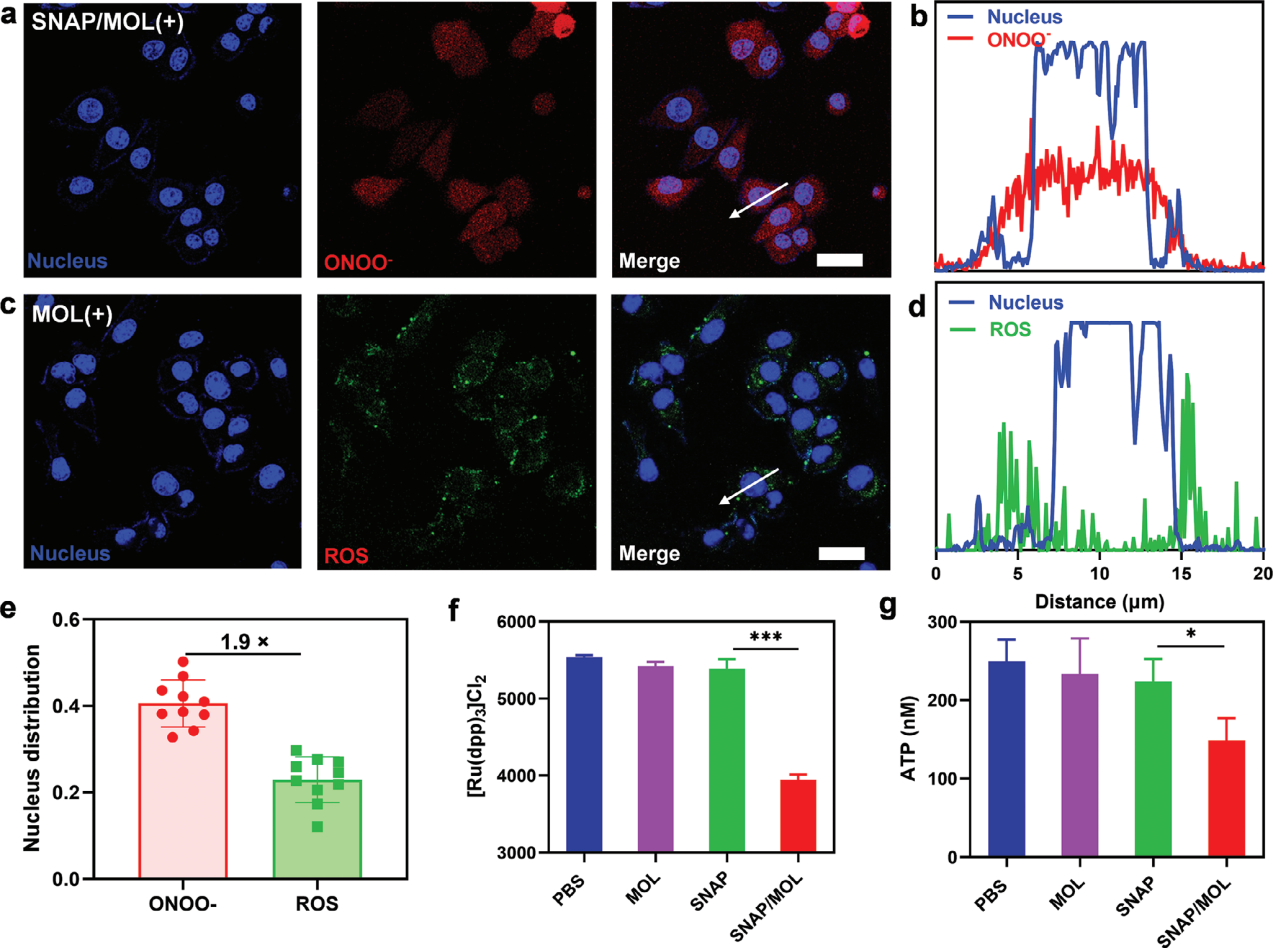
Mechanistic studies of radiosensitization of SNAP/MOL. CLSM images showing distribution of a) ONOO^−^ and b) ROS in 4T1 cells after SNAP/MOL(+) treatment. c) Colocalization between nucleus (blue) and ONOO^−^ (red) in the region of interest (ROI, white arrow in Figure 5a). d) Colocalization between nucleus (blue) and ROS (green) in the ROI (white arrow in Figure 5b). e) Relative nucleus distribution (RND) statistics for ONOO^−^ and ROS. *n* = 10. f) Flow cytometry results showing the hypoxia level of 4T1 cells after different treatments. g) Intracellular ATP levels of 4T1 cells after different treatments. f,g, data are presented as mean ± SD (*n* = 3). Statistical significance was calculated via unpaired two‐tailed student's t test. *p<0.05, ***p<0.001.

Systematic comparisons of ROS and ONOO^−^ distributions in SNAP/MOL(+)‐treated 4T1 cells showed that the ROS probed by dichlorofluorescein (DCF) were distributed in the cytoplasms while ONOO^−^ probed by DAX‐J2 sensor was evenly distributed throughout the cells (**Figure** **5**a–d). We used relative nucleus distribution (RND) index, which is defined as the ratio of the fluorescence in the nuclear region to the fluorescence of the whole cell, to quantitatively compare the intracellular distribution of ROS and ONOO^−^. ONOO^−^ showed 1.9‐fold higher RND than ROS, indicating significantly higher distribution of ONOO^−^ in the nucleus (Figure 5e). ONOO^−^ was reported to possess a much longer lifetime (t_1/2_ ≈ 1 s at pH 7.4) and a diffusion distance of up to 100 µm under physiological conditions, which is larger than the size of cancers cells.^[^ ^20^ ^]^ As a result, ONOO^−^ produced in the cytoplasm by SNAP/MOL(+) can readily diffuse into the nucleus to cause DNA damage.

As a hallmark of solid tumors, hypoxia greatly reduces the anticancer effect of radiotherapy.^[^ ^11a^ ^]^ Hypoxic cells in deep‐seated tumors have fewer DNA DSBs than normoxic cells in well‐oxygenated tumors under the same radiation dose.^[^ ^47^ ^]^ As NO is known to reduce tumor hypoxia, we investigated hypoxia alleviation by SNAP/MOL in 4T1 cells with Ru(dpp)_3_Cl_2_ as a hypoxia indicator. The luminescence of Ru(dpp)_3_Cl_2_ is greatly quenched by oxygen.^[^ ^48^ ^]^ CLSM images and flow cytometry studies showed that SNAP/MOL effectively quenched the red luminescence of Ru(dpp)_3_Cl_2_ in 4T1 cells cultured under hypoxic conditions by 31.8% compared to PBS control (Figure 5f; Figures S27, S28, Supporting Information). MOL treatment did not alleviate hypoxia, while SNAP had a negligible effect due to low cellular uptake. The attenuation of tumor hypoxia by SNAP/MOL was attributed to reduced mitochondrial respiration as a substantial reduction of intracellular adenosine triphosphate (ATP) level was observed in the SNAP/MOL group over other groups (Figure 5e). These findings indicate that SNAP/MOL achieves efficient radiosensitization by converting transient ROS into long‐lived ONOO^−^ and by alleviating hypoxia via reducing intracellular oxygen consumption.

### 2.5 In Vivo Radiosensitization of SNAP/MOL

**Figure 6:**
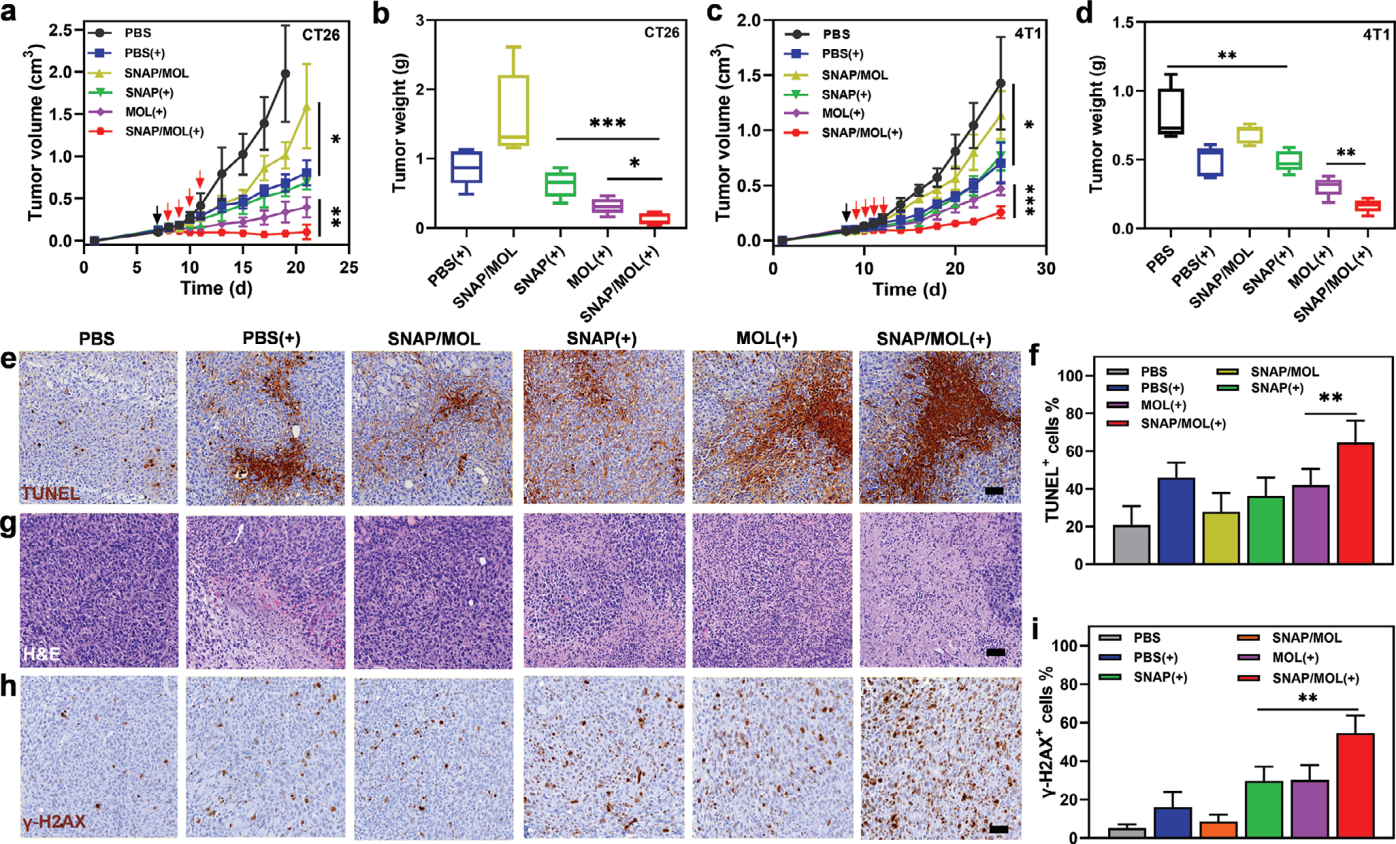
In vivo antitumor effects. a) Tumor growth profiles of CT26‐tumor‐bearing mice after different treatments. b) Resected CT26 tumor weights on day 21. c) Tumor growth profiles of 4T1‐tumor‐bearing mice with various treatment regimens. d) Resected tumor weights of 4T1 tumor‐bearing BALB/c mice at the end of therapies. e) TUNEL, g) H&E and h) γ‐H2AX staining of excised 4T1 tumors. Scale bars, 50 µm. Quantification of positive cell percentage in IHC staining of f) TUNEL and i) γ‐H2AX for evaluation of DNA damage and apoptosis. All data are shown as mean±SD, *n* = 5. Statistical significance was calculated via unpaired two‐tailed student's t test. *p<0.05, **p< 0.01, ***p<0.001.

The antitumor effects of SNAP/MOL were examined in subcutaneous CT26 murine colon cancer model and 4T1murine triple‐negative breast cancer model in BALB/c mice (Figure S31, Supporting Information). For CT26 model, SNAP/MOL moderately inhibited tumor growth with a tumor growth inhibition index (TGI) of 48.8%, while PBS(+) showed a modest antitumor effect with a TGI of 65.4% (**Figure** **6**a,b), indicating the limited antitumor effects of SNAP/MOL or radiotherapy alone. MOL(+) afforded a favorable therapeutic performance with a TGI of 82.8% while SNAP(+) did not show a noticeable advantage over PBS(+) with a TGI of 70.4%, likely due to the rapid metabolism and clearance of SNAP from tumors. In contrast, SNAP/MOL(+) synergized MOL‐mediated radiosensitization and NO‐mediated ROS conversion to arrest tumor growth with a TGI as high of 95.4% (Figures S32, S33, Supporting Information).

At a lower X‐ray dose for 4T1 model, PBS(+) and MOL(+) were less effective with TGI values of 49.6% and 66.9%, respectively (Figure 6c,d). SNAP/MOL and SNAP(+) showed TGI values of 20.2% and 46.3, respectively. In contrast, SNAP/MOL(+) was more effective with TGI of 81.2% (Figures S32, S34, Supporting Information). No obvious weight loss or major organ toxicity was observed during the treatment period for both CT26 tumor‐ and 4T1 tumor‐bearing mice (Figure S33–S35, Supporting Information), suggesting the lack of general toxicity for these treatments.

We quantitatively assessed apoptosis in 4T1 tumor tissues after the last X‐ray irradiation on day 12. After four daily fractions of 2 Gy X‐ray irradiation, SNAP/MOL(+) gave an apoptotic rate of 61.9% by flow cytometry, which is significantly higher than those of other experimental groups (Figure S38, Supporting Information). Terminal deoxynucleotidyl transferase dUTP nick end labeling (TUNEL) immunohistochemistry (IHC) staining showed significantly higher apoptosis rates in the SNAP/MOL(+) group over other groups, with 3.1‐fold more TUNEL‐positive cells than PBS control (Figure 6e,f). The hematoxylin and eosin (H&E) staining of tumor tissues exhibited severe necrosis of cancer cells after SNAP/MOL(+) treatment (Figure 6g). Ki67 staining revealed that SNAP/MOL(+) could effectively inhibit tumor proliferation (Figure S36, Supporting Information). DNA DSBs after different treatments were further evaluated by γ‐H2AX IHC staining. PBS(+), SNAP(+) and MOL(+) increased the proportion of γ‐H2AX‐positive tumor cells from 5.2% (PBS) to 16.2%, 29.6% and 30.1%, respectively (Figure 6h,i). SNAP/MOL(+) treatment further increased this proportion to 54.6%, indicating substantially more DNA damage than other groups.

### 2.6 SNAP/MOL Reduces Hypoxia and Suppresses Pulmonary Metastasis in Vivo

**Figure 7:**
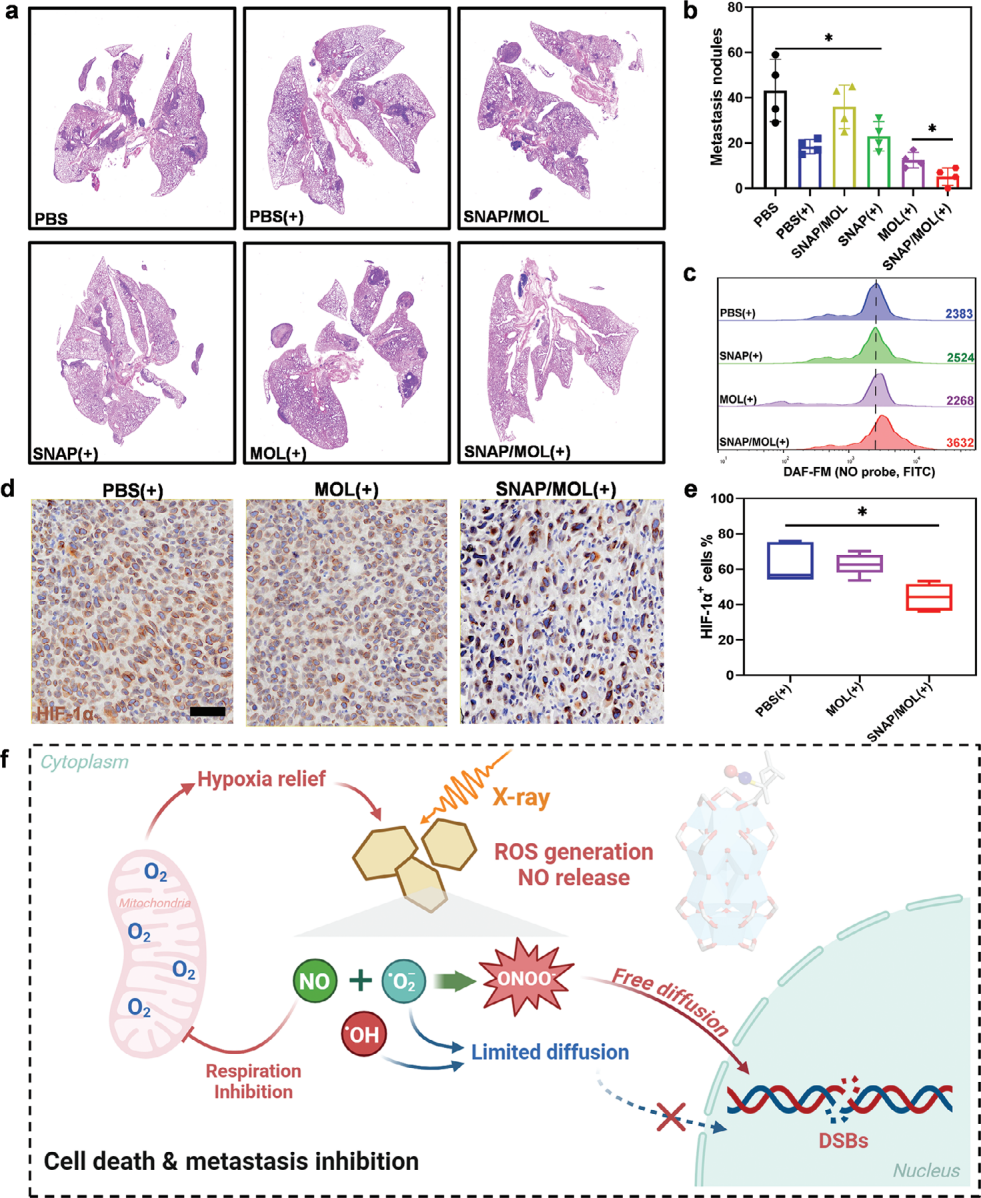
Pulmonary metastasis assessment and mechanistic summary. a) Representative images of H&E staining of lungs of 4T1‐tumor‐bearing BALB/c mice after different treatments. b) Statistical analysis of pulmonary metastatic nodules in different groups. *n* = 4. c) NO level in tumor tissues measured by CLSM using probe DAF‐FM. d) Representative IHC staining of HIF‐1α (brown) in excised 4T1 tumors after different treatments. Scale bars, 50 µm. e) Quantification of HIF‐1α positive cells by IHC staining to assess tumor hypoxia. *n* = 5. f) Schematic presentation of SNAP/MOL‐mediated radiosensitization. Under low‐dose X‐ray irradiation, SNAP/MOL releases NO and ROS in a spatiotemporally synchronized manner, leading to efficient generation of long‐lived and highly toxic ONOO^−^, thereby increasing DNA damage. Meanwhile, the sustained release of NO in the TME relieves tumor hypoxia and reduces radioresistance of tumor cells. Figure 7f was created with BioRender.com. All data are shown as mean±SD. Statistical significance was calculated via unpaired two‐tailed student's t test. *p<0.05.

Most cancer‐associated deaths occur due to metastasis.^[^ ^49^ ^]^ Because SNAP/MOL(+) induced robust immunogenic cell death (ICD) (Figure S29, Supporting Information), we evaluated its impact on cancer metastasis using 4T1 model. The anti‐migration property of SNAP/MOL(+) was first assessed by wound healing in 4T1 cells. The scratch wound assay showed that SNAP/MOL(+) inhibited wound healing and cellular invasion 2.2‐fold and 6.0‐fold more effectively than MOL(+) and PBS(+), respectively (Figure S30, Supporting Information). We next investigated the antimetastatic ability of SNAP/MOL(+) in vivo on 4T1 tumor model. The gross appearances (Figure S39, Supporting Information) and H&E sections (**Figure** **7**a) of lungs and counts of metastatic nodules (Figure 7b) revealed that the formulation of metastatic foci was effectively suppressed in SNAP/MOL(+) group, with 8.2‐, 3.5‐, 6.9‐, 4.4‐, and 2.4‐fold fewer metastatic nodules than PBS, PBS(+), SNAP/MOL, SNAP(+), and MOL(+) groups, respectively.

Hypoxia is a known microenvironment factor that promotes metastatic progression.^[^ ^50^ ^]^ We examined if the superior anti‐metastatic ability of SNAP/MOL(+) might be related to hypoxia alleviation. A significant decrease in HIF‐1α expression was observed in SNAP/MOL(+) group over PBS(+) and MOL(+) group (Figure 7d,e; Figure S40, Supporting Information), indicating a key role of MOL‐conjugated SNAP in alleviating tumor hypoxia. The reduced hypoxia in SNAP/MOL(+) group may be attributed to the sustained release of NO in tumor tissues from SNAP/MOL (Figure 7c). Taken together, our studies show that SNAP/MOL(+) not only can potently inhibit primary tumors, but also reduces distant lung metastasis by inducing immunogenicity and alleviating hypoxia.

## 3 Conclusion

As summarized in Figure 7f, the NO donor SNAP was conjugated to the Hf_12_ SBUs of Hf‐DBP‐based MOL to afford SNAP/MOL for enhanced cancer radiotherapy. Under X‐ray irradiation, SNAP/MOL not only releases NO but also efficiently produces O_2_^−.^ via transiently forming Hf^III^ centers by electrode reduction of and electron transfer from DBP excited states to Hf^IV^ centers. The spatio‐temporally synchronized generation of both NO and O_2_^−.^ facilitates the formation of long‐lived and highly cytotoxic ONOO^−^ which efficiently diffuse into the nucleus to cause increased DNA damage. In the meanwhile, the sustained release of NO from SNAP/MOL in the TME alleviates tumor hypoxia and reduces radioresistance of tumor cells. Consequently, SNAP/MOL plus low‐dose X‐ray irradiation not only inhibits the growth of both colorectal carcinoma and aggressive triple‐negative breast cancer in mouse models, but also significantly reduces distal pulmonary metastasis. This work highlights the potential of MOLs as a novel nanoplatform to mediate the generation of long‐lived and highly cytotoxic RNS and overcome tumor hypoxia and radiorestsance for highly effective cancer radiotherapy.

## 4 Experimental Section

### Materials and Methods

All chemicals for the synthesis of nanoscale metal‐organic layers (MOLs) were purchased from Sigma‐Aldrich or Fisher Scientific (USA) and used without further purification. S‐Nitroso‐N‐acetyl‐DL‐penicillamine (SNAP) was purchased from TargetMol Chemicals Inc. 5,5‐Dimethyl‐1‐pyrroline N‐oxide (DMPO) was bought from Cayman Chemical Company. Hafnium oxide nanopower (HfO_2_, 61–80 nm, cubic) was bought from US Research Nanomedicine, Inc. Dihydrorhodamine 123 (DHR123) and diaminofluorescein‐FM diacetate (DAF‐FM DA) were obtained from MedChemExpress. Cell Meter Fluorimetric Intracellular Peroxynitrite Assay Kit was purchased from AAT Bioquest Inc. Griess Reagent kit, 2′,7′‐dichlorodihydrofluorescein diacetate (DCFH‐DA), dead cell apoptosis kit with annexin V Alexa Fluor 488 & propidium iodide (PI), and hydroxyphenyl fluorescein (HPF) were purchased from Invitrogen. Cellular Superoxide Detection Assay Kit and luminescent ATP detection assay kit were purchased from Abcam. Phospho‐histone H2A.X (Ser139) (γ‐H2AX) rabbit monoclonal antibody was purchased from Cell Signaling Technology. Phosphate‐buffered saline (PBS) and RPMI‐1640 medium were obtained from Corning. Trypsin‐EDTA solution and HyClone penicillin‐streptomycin 100 × solution were purchased from Cytiva USA.

Murine colorectal carcinoma CT26 and murine triple negative breast cancer 4T1 cell lines were purchased from the American Type Culture Collection (ATCC, Rockville, MD), and cultured in RPMI‐1640 medium with 10% fetal bovine serum (FBS) and penicillin G sodium (100 U mL^−1^) and streptomycin sulfate (100 µg mL^−1^) in a humidified atmosphere with 5% CO_2_ at 37 °C. For in vivo experiments, BALB/c mice aged 6–8 weeks were used. The animal study protocol (#72 408) received approval from the Institutional Animal Care and Use Committee (IACUC) at the University of Chicago, under PHS Assurance #D16‐00322 (A3523‐01). Histology‐related services were provided by the Human Tissue Resource Center at the University of Chicago.

Dynamic light scattering (DLS) and ζ potential measurements were conducted on a Malvern Zetasizer Nano ZS. For powder X‐ray diffraction (PXRD), data were collected with a Bruker D8 Venture diffractometer utilizing Cu Kα radiation (λ = 1.54178 Å) and analyzed using PowderX software. Transmission electron microscopy (TEM) was performed using TECNAI F30 HRTEM instrument. Atomic force microscopy (AFM) images were obtained with the Bruker Multimode 8‐HR instrument. UV–vis spectra were measured with a Shimadzu UV‐2600 spectrophotometer. Inductively coupled plasma‐mass spectrometry (ICP‐MS) data were acquired with an Agilent 7700x ICP‐MS and processed using ICP‐MS Mass Hunter version 4.6 C.01.06. Samples were prepared in a 2% HNO_3_ matrix and analyzed with ^159^ Tb and internal standards over a 10‐point standard curve ranging from 1 ppb to 500 ppb, achieving a correlation of *R* > 0.999. ^1^H NMR spectra were recorded on a Bruker NMR 400 DRX spectrometer operating at 400 MHz, with references to the proton resonance of CDCl_3_ (δ = 7.26) or DMSO‐d6 (δ = 2.50). X‐ray photoelectron spectroscopy (XPS) was performed on the KRATOS AXIS NOVA X‐ray Photoelectron Spectrometer, based on a monochromatic Al Kα X‐ray source at X‐ray Research Facilities of University of Chicago. The electron paramagnetic resonance (EPR) signal was collected by a Bruker Elexsys 500 X‐band EPR.

Flow cytometry was performed using an LSR‐Fortessa 4–15 (BD Biosciences, USA) and analyzed with FlowJo software (Tree Star, USA). The absorbance and fluorescence measurements from well plates were performed on a BioTek Synergy HTX microplate reader. Confocal laser scanning microscopy (CLSM) images were captured with a Leica Stellaris 8 at the University of Chicago Integrated Light Microscopy Facility, with analysis conducted using ImageJ software (NIH, USA). Live cell imaging was performed with an IncuCyte S3 at the Cellular Screening Center at the University of Chicago. Histological slides were scanned using a CRi Pannoramic SCAN 40 × whole slide scanner, with analysis performed using QuPath software. For test tube and in vitro X‐ray irradiation experiments, a Philips RT250 orthovoltage X‐ray machine (USA) was employed, set at 250 kVp, 15 mA, and equipped with a 1 mm Cu filter. For animal irradiation, an X‐RAD 225 image‐guided biological irradiator (Precision X‐ray Inc., USA) was used, operating at 225 kVp, 13 mA, with a 0.3 mm Cu filter and a 15 mm collimator. The X‐ray dose rate of the X‐RAD 225 was 0.0 4167 Gy/second. Both X‐ray instruments were routinely calibrated for dosimetry using an ionization chamber by the Department of Radiation Oncology at the University of Chicago.

### Synthesis and Characterization of MOL and SNAP/MOL

5,15‐di(p‐benzoato)porphyrin (H_2_DBP) and Hf‐DBP MOL were synthesized according to a previous report.^[^ ^24^, ^51^ ^]^ In brief, Hf‐DBP was prepared by combining 2 mg of HfCl_4_, 1 mg of H_2_DBP, 8.5 µL of propionic acid (PA), 5 µL of water, and 1 mL of DMF in a one‐dram vial. This mixture was then heated at 80 °C for 24 h. The resulting purple particles were separated by centrifugation, washed with DMF and ethanol, and subsequently stored in ethanol as suspensions. Hf‐DBP MOF (MOF) was synthesized according to a previous report with its representative TEM image shown in Figure S2 (Supporting Information).^[^ ^7a^ ^]^

Trifluoroacetic acid (TFA)‐modified Hf‐DBP MOL (MOL) was synthesized as previously reported.^[^ ^26^ ^]^ An ethanol suspension of Hf‐DBP was washed with anhydrous acetonitrile and toluene. The suspension was degassed with N_2_. Next, 5 mL of Hf‐DBP in toluene (with a DBP concentration of 1 mM) was added and treated with a 20‐fold excess of trimethylsilyl trifluoroacetate (TFA‐TMS) relative to propionic acid (PA). The reaction mixture was stirred at room temperature for 16 h. Afterwards, MOL was collected by centrifugation and sequentially washed with acetonitrile and ethanol. To prepare SNAP/MOL, 1 mL MOL was dispersed in ethanol at a DBP concentration of 1 mM. 4.9 mg of SNAP was added to the suspension and stirred at room temperature for 5 h. The resulting SNAP/MOL was collected by centrifugation, washed with ethanol, and stored in ethanol in a 4 °C refrigerator. The loading amount was quantified by ^1^H NMR spectroscopy.

For NMR studies, 1 mg of MOL or SNAP/MOL suspensions was centrifuged and dried under vacuum and then digested in a solution of 500 µL D_6_‐DMSO, 50 µL D_2_O, and 50 µL D_3_PO_4_ by sonication for 10 min. The mixture was then analyzed by ^1^H‐NMR. The H_2_DBP concentration was also determined by UV–vis spectroscopy (Figure S3, Supporting Information).

### ·OH Detection

The generation of ·OH under irradiation was assessed using the APF assay. APF was added to PBS suspensions of MOL, with a Hf concentration of 40 µM. The final APF concentration in the mixture was 5 µM. For the assay, 100 µL of each suspension was placed in a 96‐well plate (*n* = 3) and irradiated with X‐rays at doses of 0, 2, 4, 8, 16 Gy. Fluorescence signals (Em, 520 nm) were measured using a Synergy HTX microplate reader with an excitation wavelength of 485 nm.

### O_2_^−^^.^ Detection

DHR123 probe was employed to detect O_2_^−.^ production under X‐ray irradiation. MOF or MOL was dispersed in ultrapure water at the same Hf concentration of 40 µM, followed by the addition of the DHR123 probe to give a final concentration of 10 µM. The fluorescence intensities of the dispersions were quickly detected after different doses of X‐ray irradiation (0, 2, 4, 8, 16 Gy) using Synergy HTX microplate reader and a fluorescence spectrometer (Ex 485 nm, Em 520 nm). The production of O_2_^−.^ by MOL at different concentrations under X‐ray irradiation was detected using a similar approach. For O_2_^−.^ quenching assay, 50 µM ascorbic acid (Vc) or 50 µg mL^−1^ superoxide dismutase (SOD) was added to the above aqueous solution prior to X‐ray irradiation. DHR123 aqueous solution without the MOF or MOL was used as a control.

### EPR Test

Electron paramagnetic resonance (EPR) was used to detect O_2_^−.^ formation. Briefly, MOL was suspended in methanol with a Hf concentration of 200 µM in the presence of 100 mM DMPO. A methanol solution of 100 mM DMPO was used as control. The EPR signal was immediately collected with a Bruker Elexsys 500 X‐band EPR after irradiation with 8 Gy X‐rays. Hf^III^H‐DBP MOL was synthesized by reduction with NaBEt_3_H.^[^ ^33^ ^]^ In a N_2_‐filled glove box, MOL (1.5 µmol of Hf) was charged to a 1.5 mL centrifuge tube and dispersed in 0.5 mL of toluene. NaBEt_3_H (15 µL, 1.0 M solution in toluene) was then added dropwise to the suspension. The color of the MOL changed immediately from purple to brown while vigorously evolving H_2_ gas. The resulting suspension was kept at room temperature for 2 h to ensure complete reduction. The brown solid was then centrifuged out of suspension and washed 3 times with toluene to remove excess NaBEt_3_H and side products (NaCl and BEt_3_). After treatment with oxygen, the EPR signal was immediately acquired at 20 K.

### NO Release in Test Tubes

NO can easily convert to nitrite, which reacts with the Griess regent to form a diazo compound with strong absorbance at 540 nm. SNAP/MOL was suspended in an aqueous solution with a SNAP concentration of 200 µM. After GSH incubation and X‐ray irradiation (8 Gy), freshly prepared Griess reagent was added to each well. The mixture was further incubated at room temperature for 30 min, and the absorbances (540 nm) were measured using a Synergy HTX microplate reader. The standard curve for NO was shown in Figure S12 (Supporting Information)

### Intracellular Detection of O_2_^− ·^

4T1 cells were cultured in 35 mm glass bottom dishes (1 × 10^5^ cells per well) until adherence. SNAP, MOL, or SNAP/MOL was added at a Hf concentration of 40 µM or/and a SNAP concentration of 9.6 µM and incubated for 8 h. The cells were then incubated with Superoxide Detection Reagent (1 µM) for 1 h, followed by X‐ray irradiation at a dose of 4 Gy. After that, the cells were washed twice with PBS and incubated with Hoechst 33 342 (5 µM) for 15 min. After washing twice with warm PBS, the cells were imaged on a Leica Stellaris 8 microscope. O_2_^−.^ generation was also quantified by flow cytometry using the FITC channel.

### Intracellular ·OH Detection

The ·OH generation by SNAP, MOL, or SNAP/MOL plus X‐ray irradiation was evaluated on 4T1 cells by CLSM and flow cytometry. For flow cytometry analysis, 4T1 cells were plated on 6‐well plates at a density of 2 × 10^5^ cells/well. The cells were then treated with SNAP, MOL, or SNAP/MOL at a DBP concentration of 20 µM and incubated for an additional 8 h. Following the treatments, 20 µM HPF was added to each well and the cells were incubated for one additional hour. The plates were then irradiated with X‐ray at a dose of 4 Gy. After washing with PBS twice, the cells were scraped off and analyzed by flow cytometry. For CLSM, 4T1 cells were seeded in 35 mm glass‐bottom dishes (1 × 10^5^ cells/well) and cultured overnight. The cells were treated similarly as above but were not detached. After washing three times with PBS, the medium was replaced with warm medium, and imaging was performed immediately using a Leica Stellaris 8 microscope.

### Measurement of Intracellular NO Release

DAF‐FM DA was used to detect low concentrations of NO. DAF‐FM DA spontaneously crosses cell membranes and was subsequently cleaved by esterases to produce DAF‐FM intracellularly. DAF‐FM reacts with NO to form fluorescent benzotriazole (Ex/Em = 495/515 nm). Briefly, 4T1 cells were cultured in 35 mm glass‐bottom dishes (1 × 10^5^ cells per well) until adherence. Afterwards, the cells were treated with SNAP, MOL, SNAP/MOL, or SNAP plus MOL for 8 h with a DBP concentration of 20 µM. The medium was removed and then the cells were incubated with fresh medium containing 10 µM DAF‐FM‐DA for 1 h. The cells were washed twice with PBS and then subjected to X‐ray irradiation (4 Gy). After staining with Hoechst 33 342 (5 µM) for 15 min, the cells were imaged on a Leica Stellaris 8 microscope. For flow cytometric analysis, 4T1 cells seeded in 6‐well plates were subjected to the same treatments and the cells were scraped off to quantify the fluorescent signals of DAF‐FM by flow cytometry.

### ONOO^−^ Detection in Vitro

DAX‐J2 PON was employed to probe ONOO^−^ in cancer cells as it can specifically react with intracellular ONOO^−^ to generate a product with bright green fluorescence. 4T1 cells were seeded in 35 mm glass‐bottom dishes at a density of 1 × 10^5^ cells/well. After incubation for 12 h, the cells were incubated with SNAP, MOL, SNAP/MOL, and SNAP plus MOL for 8 h at a DBP concentration of 20 µM. The cell medium was then replaced with fresh one containing DAX‐J2^TM^ PON (1 ×). After culturing for 1 h in the dark, all treated cells were gently washed with PBS and then observed by CLSM. Flow cytometry was also used to quantify the ONOO^−^ content after different treatments.

### Cellular Uptake

The cellular uptake of MOL and SNAP/MOL was evaluated in 4T1 cells by flow cytometry and ICP‐MS. The cells were seeded in 6‐well plates at a density of 2 × 10^5^ cells/well. MOL or SNAP/MOL was added at a DBP concentration of 10 µM. The cells were then incubated at 37 °C in an incubator for 1, 2, 4, 8 and 12 h. At each time point, the cells were washed three times with PBS, digested, counted, and centrifuged to obtain a cell precipitate. After digestion of the cells with 1 ml of nitric acid containing 1% hydrofluoric acid, the Hf content in the digest was measured by ICP‐MS to determine cellular uptake. With the same treatments, the cellular uptake of SNAP/MOL was also assessed via flow cytometry by measuring DBP fluorescence at different time points.

### Clonogenic Assay

4T1 cells were seeded in 6‐well plates (1 × 10^5^ cells per well) and cultured until adherence. The cells were then incubated with PBS, MOL, or SNAP/MOL at a Hf concentration of 60 µM for 6 h, followed by X‐ray irradiation with dose of 0, 2, 4, 6, 8, 10 Gy. The irradiated cells were trypsinized and counted, and then re‐seeded in 6‐well plates at a density of 200 cells per well. After incubation in for 7 more days, the cells were gently washed with PBS and fixed with 4% paraformaldehyde for 20 min. The plates were rinsed twice with PBS and then stained with 500 µl of 0.5% crystal violet (50% methanol / H_2_O). The plates were gently rinsed with water 3 times and the colonies were counted with Image J software. The dose modifying ratio at 10% survival fraction (DMR_10%_) was used as a parameter for the radiosensitization effect and defined as the dose ratio that produces 10% survival fraction in the control and experimental groups.^[^ ^29b^ ^]^

### In Vitro Apoptosis Analysis

The apoptosis under X‐ray irradiation was evaluated in 4T1 cells by flow cytometry. 4T1 cells were seeded in 6‐well plates and incubated until adherence. After which, the medium was replaced by fresh medium containing SNAP, MOL, or SNAP/MOL at a DBP concentration of 40 µM and cultured for 8 additional hours. Thereafter, the cells were irradiated with 4 Gy X‐ray. 24 hours later, the cells were washed with PBS and trypsinized to obtain single‐cell suspensions. The cells were then stained using a dead cell apoptosis kit with Annexin V Alexa Fluor 488 and PI according to the manufacturer's instructions. The stained cells were resuspended in binding buffer for flow cytometric analysis (Annexin V: FITC channel, PI: PE‐dazzle 594 channel).

### γ‐H2AX Staining for CLSM and Flow Cytometry Analysis

4T1 cells were plated at a density of 1 × 10^5^ cells/well in 6‐well plates and incubated overnight. The medium was replaced by fresh medium containing SNAP, MOL, or SNAP/MOL at a DBP concentration of 40 µM and the cells were cultured for 24 additional hours. For CLSM imaging, the cells were first washed with PBS and then fixed with 4% paraformaldehyde at room temperature for 20 min. After rinsing with PBS, the cells were blocked and permeabilized with 5% FBS and 0.3% Triton‐X in PBS at room temperature for 1 h. Following blocking, the cells were incubated with γ‐H2AX primary antibody (1:500) in 1% BSA and 0.3% Triton‐X in PBS at room temperature for 1 h. The cells were then washed with PBS and incubated with Alexa Fluor 488‐conjugated secondary antibody (1:3000) in 1% BSA and 0.3% Triton‐X in PBS at room temperature for 1 h. After another wash with PBS, the cells were incubated with Hoechst 33 342 (5 µM) in PBS at 37 °C for 15 min to stain the nucleus. Finally, the cells were washed with PBS three times and examined using a Leica Stellaris 8 confocal microscope. The obtained images were processed and analyzed by Fiji ImageJ (NIH). For flow cytometry, the cells were prepared as single‐cell suspensions and underwent fixation, blocking, and permeabilization using the same procedures as described earlier. The cells were stained with primary and secondary antibodies and suspended in 0.5% BSA in PBS and analyzed by flow cytometry.

### Western Blot (WB)

After treatment in the same way as above (for DNA damage evaluation), the cells were lysed using RIPA buffer supplemented with a protease and phosphatase inhibitor cocktail according to the manufacturer's instructions. The proteins in the supernatant were collected by centrifugation at 14,000 g, and protein concentrations were determined and normalized using a BCA assay. The proteins were denatured and reduced using NuPAGE LDS sample buffer containing 50 mM DTT, and then heated at 95 °C for 10 min. Samples ranging from 10 to 20 µg were loaded onto a 4−12% NuPAGE Bis‐Tris gel and subjected to electrophoresis using an XCell SureLock Mini‐Cell at 200 V for 45 min. The proteins were then transferred to a PVDF membrane at 300 mA for 100 min using a Mini Trans‐Blot Electrophoretic Transfer Cell. The membrane was blocked at room temperature for 30 min with TBST containing 5% nonfat dry milk, and subsequently incubated overnight at 4 °C with a primary antibody (Phosphohistone H2A.X (Ser139) (20E3) rabbit mAb #9718, 1:2000) solution in TBST with 5% BSA. The membrane was washed with TBST and then incubated at room temperature for 1 hour with a secondary antibody conjugated to HRP, diluted in TBST containing 5% BSA. Following this, the membrane was washed once more with TBST, and Pierce ECL Western blotting substrate was applied. The chemiluminescent signal was subsequently detected using a FluorChem R system.

### Distribution of ONOO^−^ and ROS in 4T1 cells

4T1 cells were seeded in 35 mm glass‐bottom dishes at a density of 1 × 10^5^ cells/dish and cultured overnight. The cells were treated with MOL or SNAP/MOL at a DBP concentration of 20 µM for 8 h. After that, the cells were stained with DCFH‐DA (ROS probe) or DAX‐J2 PON (ONOO^−^ probe) for 1 h. The cells were then irradiated with 6 Gy X‐ray. After rinsing with warm PBS twice, the cell nuclei were stained with Hoechst 33 342. The distribution of ONOO^−^ and ROS in 4T1 cells were observed by Leica Stellaris 8 microscope. The relative nucleus distribution index (RND) was defined as:

(1) $$\mathrm{RND}=\frac{\mathrm{Flu}\;\mathrm{of}\;\mathrm{ROS}\;\mathrm{or}\;\mathrm{ONO}O^{-}\;\mathrm{in}\;\mathrm{nucleus}}{\mathrm{Flu}\;\mathrm{of}\;\mathrm{ROS}\;\mathrm{or}\;\mathrm{ONO}O^{-}\;\mathrm{in}\;\mathrm{whole}\;\mathrm{cell}}\times 100\%$$

### Intracellular Oxygen Detection

Ru(dpp)₃Cl₂ was employed as a hypoxia probe due to its ability to luminesce in response to low oxygen levels. 4T1 cells were seeded in 35 mm glass‐bottom dishes at a density of 1 × 10^5^ cells/well and incubated until adherence. The medium was replaced with fresh one containing PBS, SNAP, MOL or SNAP/MOL at a DBP concentration of 30 µM. The cells were transferred to a hypoxia chamber (pO_2_ = 0.5) and cultured for 24 h. The cells were washed with PBS twice and stained with Ru(dpp)₃Cl₂ (10 µg mL^−1^) in a hypoxia chamber for 1 h. Cellular hypoxia was observed on a Leica Stellaris 8 microscope after staining with Hoechst 33 342 (5 µM). In addition, the fluorescence intensity of Ru(dpp)_3_Cl_2_ after different treatments was quantified by flow cytometry.

### Intracellular ATP Measurement

4T1 cancer cells were seeded in 6‐well plates and cultured until adherence. After treatment with SNAP, MOL or SNAP/MOL at a DBP concentration of 30 µM for 24 h, the cells were rinsed with PBS twice and lysed with RIPA buffer. The cellular ATP content was determined with luminescent ATP assay kit according to the manufacturer's instructions (Invitrogen, USA). The measurements were normalized by cell number and the experiments were performed three times.

### CRT Exposure

For flow cytometry analysis, 4T1 cells were seeded in 6‐well plates at a density of 2 × 10^5^ cells per well and incubated overnight. The cells were treated with SNAP, MOL, or SNAP/MOL at a Hf concentration of 40 µM for 8 h. The cells were irradiated with X‐ray (6 Gy) and then incubated at 37 °C for 24 h. The cells were stained with Alexa Fluor 488‐conjugated anti‐CRT (NOVUS, diluted 1:100) for 30 minutes, washed twice with PBS, and subsequently analyzed using flow cytometry. For CLSM analysis, 4T1 cells were plated at a density of 1 × 10^5^ cells per well in 35 mm glass‐bottom dishes and cultured overnight. The cells were then treated in the same way as for flow cytometric study. Afterward, the cells were fixed with cold methanol for 5 min and stained overnight with Alexa Fluor 488‐conjugated anti‐Calreticulin (NOVUS, diluted 1:100) in 2% BSA at 4 °C. Following this, the cells were stained with Hoechst 33 342 (diluted 1:3000) in PBS for 15 min. After washing twice with PBS, the cells were observed under a Leica Stellaris 8 microscope.

### ATP Secretion

4T1 cells were seeded in 6‐well plates at a density of 2 × 10^5^ cells per well and cultured overnight. The cells were then treated with SNAP, MOL, or SNAP/MOL at a Hf concentration of 40 µM for 8 h. The cells were irradiated with 6 Gy X‐ray and incubated at 37 °C for 24 additional hours. Subsequently, the supernatants were collected and analyzed for ATP content using an ATP detection kit (Invitrogen, USA) according to the manufacturer's instructions.

### Wound Healing Assay

To assess the invasion and migration capabilities of 4T1 cells, a wound healing assay was conducted. The cells were seeded at a density of 5 × 10^4^ cells per well in a 96‐well plate and cultured overnight. A wound was then created using an Incucyte 96‐well woundmaker tool. Following the wound creation, the cells were washed twice with PBS. SNAP, MOL, or SNAP/MOL at a Hf concentration of 40 µM was added to the wells and incubated for 4 h (*n* = 3). The cells were then irradiated with X‐ray at a dose of 6 Gy. Subsequently, the cells were placed in an IncuCyte S3 for live imaging over 24 h, and the data were analyzed using the scratch wound analysis module.

### In Vivo Antitumor Efficacy

In vivo anti‐cancer efficacy of SNAP/MOL was tested on subcutaneous colorectal (CT26) and triple‐negative breast (4T1) mouse models. 4T1 (2 × 10^6^) or CT26 (2 × 10^6^) cells were subcutaneously inoculated into the right flanks of BALB/c mice. When the tumors reached 75–100 mm^3^ in volume for 4T1 model or 100–130 mm^3^ for CT26 model on day 8 and day 7, respectively, the mice were randomly divided into 6 groups (*n* = 5) and subjected to different treatments: PBS, PBS(+), SNAP/MOL, SNAP(+), MOL(+), SNAP/MOL(+). The mice were injected intratumorally with a Hf dose of 1 µmol (a SNAP dose 0.24 µmol). Twelve hours after the injection, the mice were anesthetized using 2% (v/v) isoflurane. The tumors were then irradiated with 4 daily fractions of X‐rays at 2 Gy fraction for 4T1 tumors and 3 Gy/fraction for CT26 tumors. Tumor sizes were assessed using an electronic caliper (tumor volume (V) = length (L) × width (W)^2^ / 2), and body weight was tracked with an electronic scale. At the end of treatments, the mice were euthanized, and their tumors were removed and weighed. Major organs were then sectioned and stained with H&E to assess general toxicity. The tumor growth inhibition index (TGI) was calculated using the following formula:

(2) $$\mathrm{TGI}=\left(1-\frac{M_{exp}}{\bar{M_{con}}}\right)\times 100\%$$

where $\bar{M_{con}}$ and *M_exp_* represent average tumor volume of control mice and treated mice at the endpoint, respectively.

### In vivo Apoptosis and NO Generation Detection

After the last X‐ray irradiation on day 12, the 4T1 tumors were harvested. The tumors were incubated in RPMI‐1640 containing 10% FBS, 1 mg mL^−1^ of collagenase I (Gibco), 250 µg mL^−1^ of collagenase IV (Gibco), and 50 µg mL^−1^ of DNase I (Sigma‐Aldrich) at 37 °C for 1 hour. After digestion, the mixture was gently triturated and passed through sterile cell strainers (40 µm, Corning) to obtain single‐cell suspensions. The cells were washed twice with cold FACS buffer and co‐stained with Annexin V Alexa Fluor 488 and PI in binding buffer for 30 min in the dark. After rinsing with PBS, cell apoptosis was detected by flow cytometry. The single‐cell suspensions were stained with DAF‐FM DA to detect the NO content in tumor tissues after different treatments.

### Pulmonary Metastasis Evaluation

For lung metastasis assay, 4T1 tumor‐bearing mice were subjected to the same treatment as the antitumor experiments (*n* = 4). On day 26, all mice were sacrificed, and lung tissues were collected, fixed in Bouin's solution. One day after fixing, the pulmonary nodules were counted and recorded. Next, the fixed lungs were sliced and stained with hematoxylin and eosin for immunohistochemical examination.

### Immunohistochemistry Analysis

One day after the last X‐ray irradiation on day 12, the mice were euthanized and the tumors were removed. The tumor tissues were first fixed in 4% PFA for 1 day and then in 70% ethanol for another day. The tissues were embedded in paraffin, sectioned, and stained for γ‐H2AX, Ki67, HIF‐1α and TUNEL by the Human Tissue Resource Center at the University of Chicago. The slides were sealed and scanned using a CRi Panoramic SCAN 40x whole slide scanner by the Integrated Light Microscopy Core at the University of Chicago. The images were examined and analyzed using QuPath‐0.5.1 software.

### Statistical Analysis

Statistical analysis was performed using GraphPad Prism (version 8.3.0). Results were expressed as means ± standard deviation (S.D.). To determine statistical significance, unpaired two‐sided Student's t test was used for comparisons between two groups, and one‐way ANOVA was employed for comparisons involving multiple groups. Statistical significance was denoted as follows: *p<0.05, **p<0.01, ***p<0.001, and ns indicates not significant.

## Conflict of Interest

The authors declare no conflict of interest.

## Supporting information



Supporting Information

## Data Availability

The data that support the findings of this study are available from the corresponding author upon reasonable request.
