# Supplementary material for: Nitric Oxide‐Releasing Nanoscale Metal‐Organic Layer Overcomes Hypoxia and Reactive Oxygen Species Diffusion Barriers to Enhance Cancer Radiotherapy
Source: Adv Sci (Weinh). 2025 Jan 1;12(8):2413518. doi: 10.1002/advs.202413518 (PMC11848595; doi:10.1002/advs.202413518)
Supplement: Supplementary file 1 — Supporting Information [file ADVS-12-2413518-s001.pdf]

ADVANCED SCIENCE
Open Access

Supporting Information

for *Adv. Sci.*, DOI 10.1002/advs.202413518

Nitric Oxide-Releasing Nanoscale Metal-Organic Layer Overcomes Hypoxia and Reactive Oxygen Species Diffusion Barriers to Enhance Cancer Radiotherapy

*Yuxuan Xiong, Jinhong Li, Xiaomin Jiang, Wenyao Zhen, Xin Ma and Wenbin Lin**

Supporting Information

Nitric Oxide-Releasing Nanoscale Metal-Organic Layer Overcomes Hypoxia and Reactive Oxygen Species Diffusion Barriers to Enhance Cancer Radiotherapy

*Yuxuan Xiong, Jinhong Li, Xiaomin Jiang, Wenyao Zhen, Xin Ma, Wenbin Lin**

Y. Xiong, J. Li, X. Jiang, W. Zhen, X. Ma, W. Lin

Department of Chemistry

The University of Chicago

Chicago, IL 60637, USA

E-Mail: wenbinlin@uchicago.edu

W. Lin

Department of Radiation and Cellular Oncology and the Ludwig Center for Metastasis Research

The University of Chicago

Chicago, IL 60637, USA

Supplementary Figures

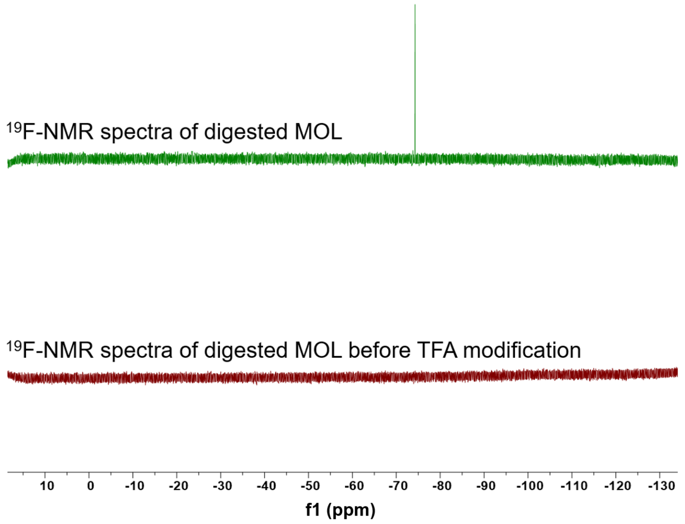

Figure S1. ^{19}F-NMR spectra of the digested MOL before and after TFA modification.

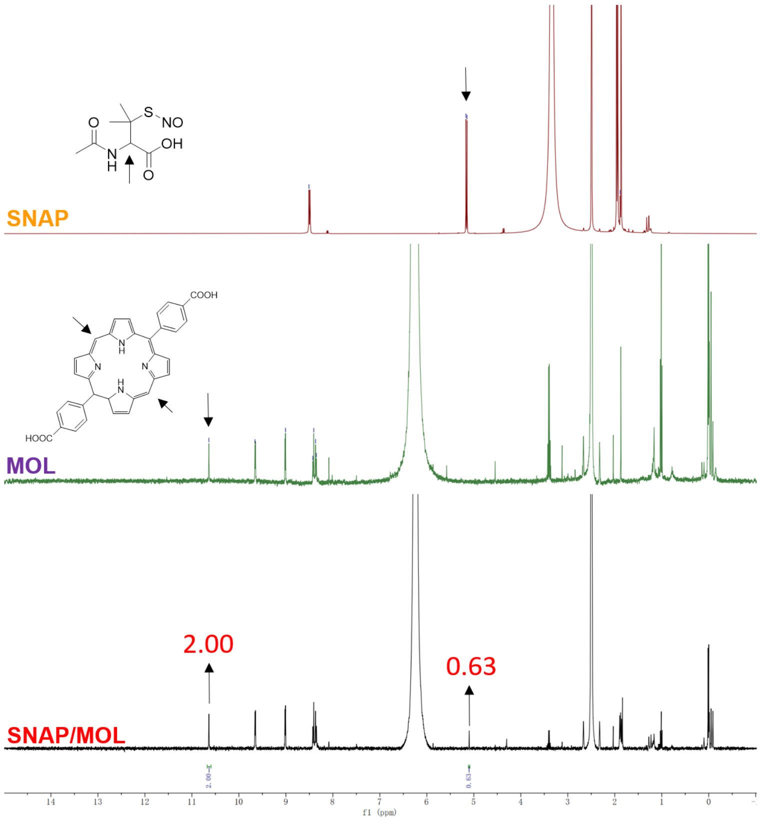

Figure S2. ^{1}H-NMR spectra of SNAP, digested MOL, and digested SNAP/MOL.

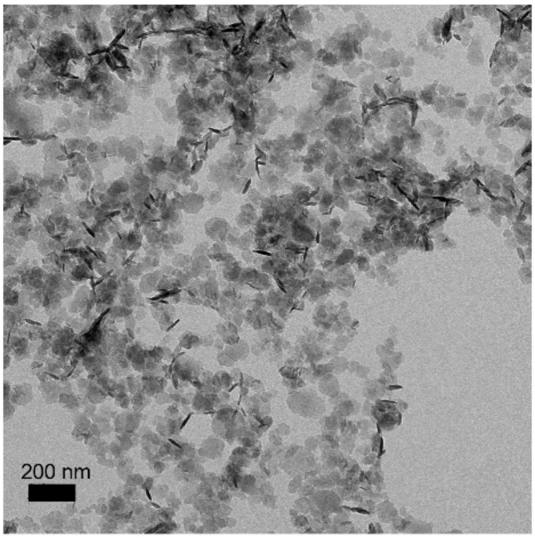

Figure S3. TEM image of three dimensional MOF.

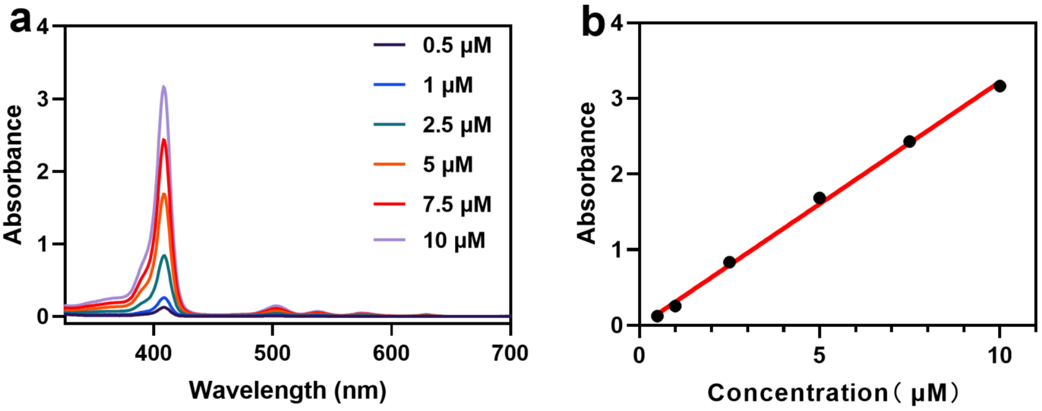

Figure S4. (a) UV-Vis absorption spectra of H_2DBP at various concentrations in DMSO with 10% H_3PO_4. (b) The linear fit of H_2DBP absorbance at 408 nm as a function of concentration.

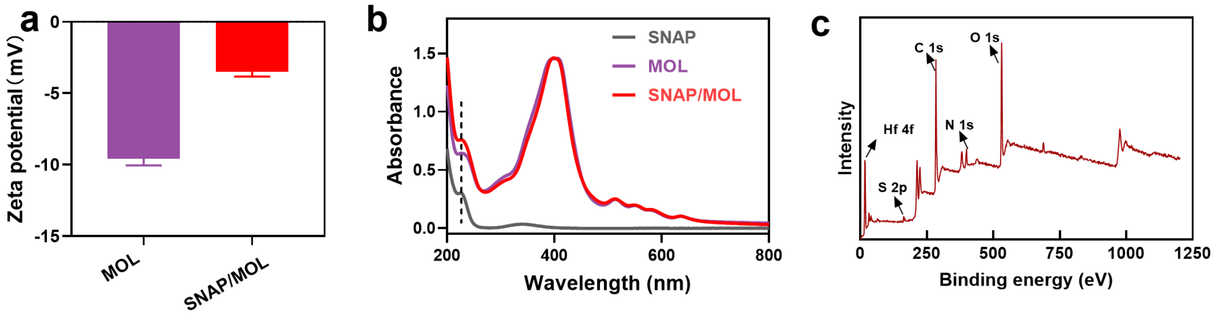

Figure S5. (a) Zeta potential values of MOL and SNAP/MOL (n=5, data are shown as mean± SD). (b) UV-Vis spectrum of SNAP, MOL and SNAP/MOL. (c) XPS spectrum of SNAP/MOL.

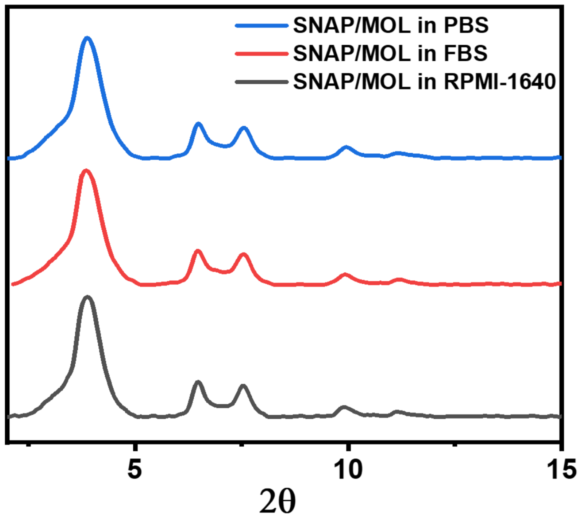

Figure S6. PXRD patterns of SNAP/MOL after incubation in biologically relevant media.

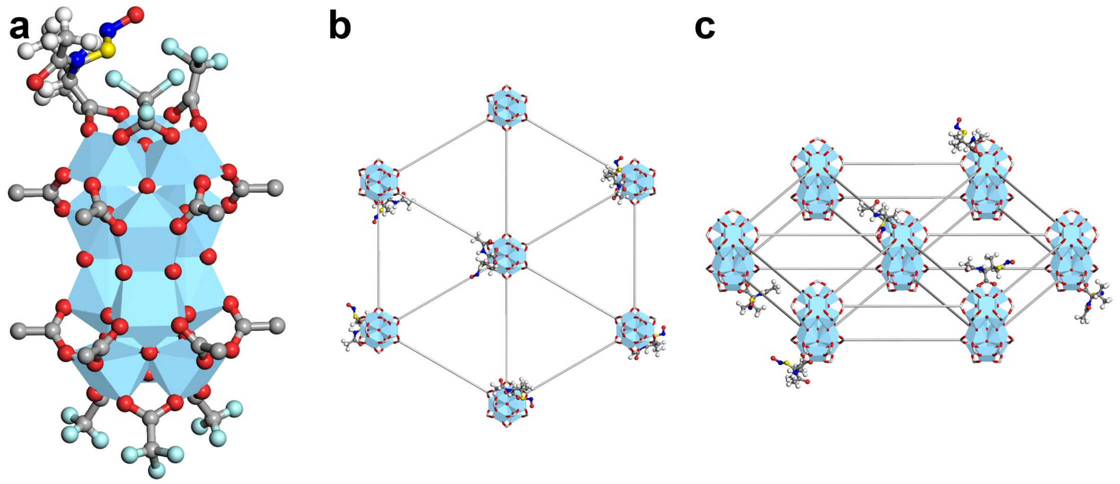

Figure S7. Structure models of SNAP/MOL. (a) The structure of mono-substituted $Hf_{12}(\mu_3\text{-}O)_8(\mu_3\text{-}OH)_8(\mu_2\text{-}OH)_6(DBP)_6(\mu_2\text{-}TFA)_{2.4}(\mu_2\text{-}SNAP)_{3.6}$. (b) The top view of SNAP/MOL reveals its monolayer structure. (c) Side view of SNAP/MOL. Light blue: Hf; red: O; navy blue: N.

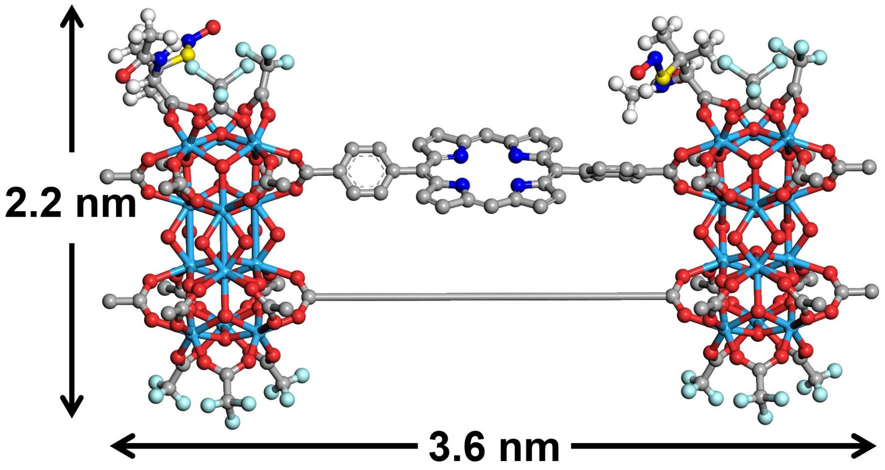

Figure S8. Size information and topological structure of SNAP-Hf_{12}-DBP unit.

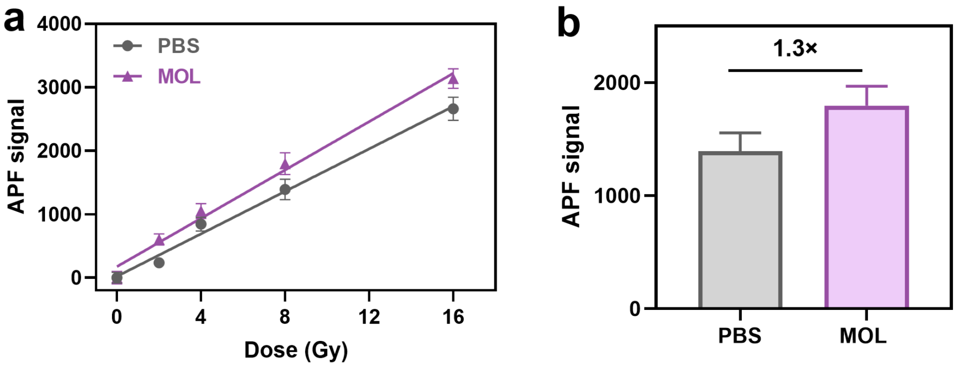

Figure S9. (a) Hydroxyl radical signal of PBS and MOL by APF assay after different doses of X-ray. (b) Fluorescence intensity of APF at 528 nm after 8 Gy X-ray irradiation (n=3). All data are shown as mean±SD.

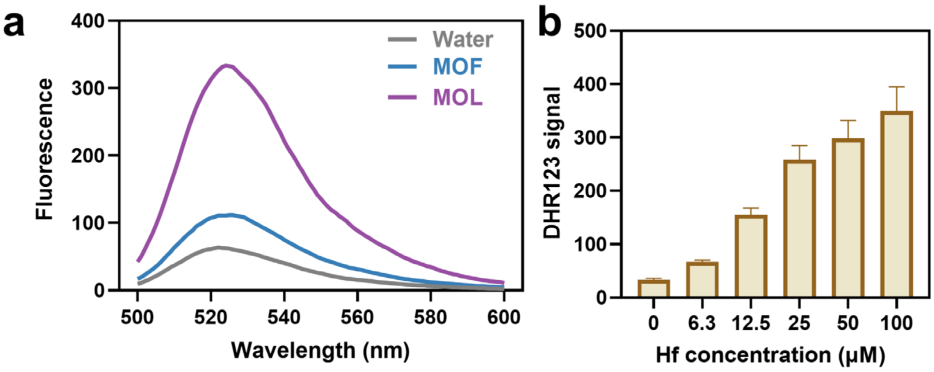

Figure S10. (a) Superoxide generation by MOL and MOF (40 μM Hf) under the irradiation of 8 Gy X-ray, using DHR123 as probe. (b) DHR123 signals of different concentrations of MOL under X-ray radiation (n=3, data are shown as mean±SD).

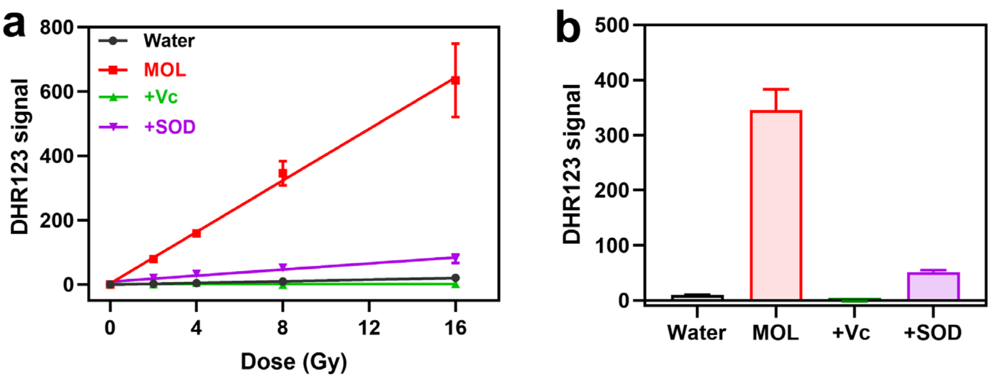

Figure S11. (a) DHR123 signals of MOL co-incubated with SOD (50 μg/mL) or Vc (50 μM) at different doses of X-ray irradiation. (b) DHR123 fluorescence intensities at 528 nm after 8 Gy X-ray irradiation. All data are shown as mean±SD, n=5.

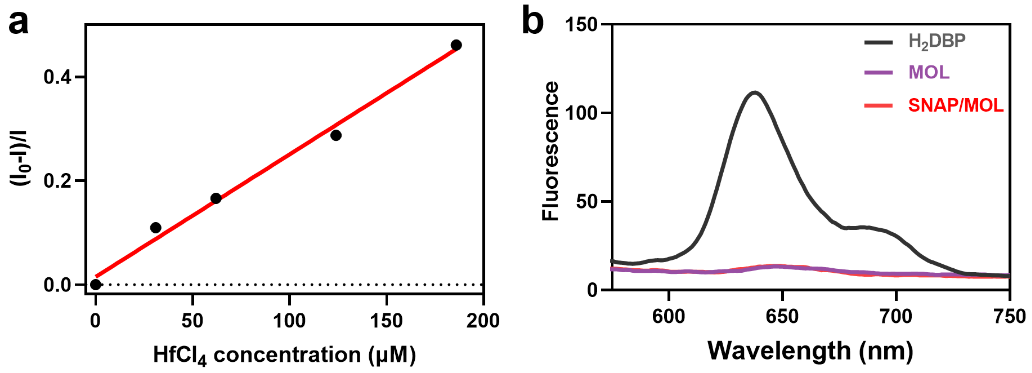

Figure S12. (a) Plots of $(I_0$-I)/I as a function of the concentration of $HfCl_4$. (b) Fluorescence of H_2DBP, MOL and SNAP/MOL with a DBP concentration of 20 μM (Ex=405 nm).

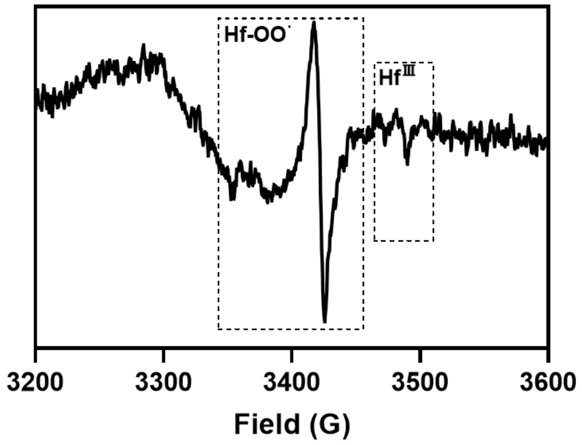

Figure S13. EPR spectrum of Hf($O_2^{\cdot}$)-DBP, showing strong superoxide and Hf^{III} signal.

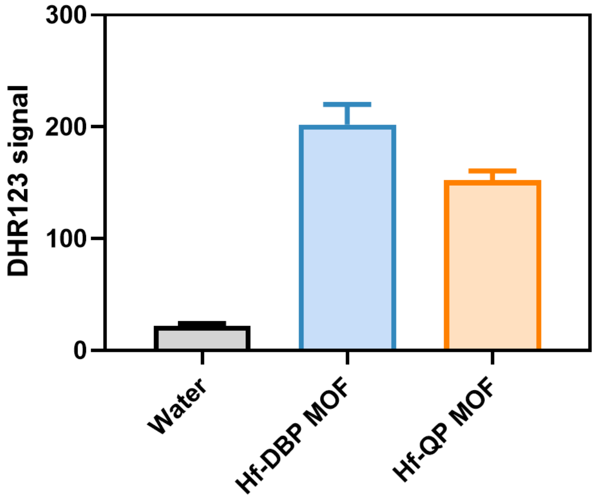

Figure S14. Superoxide generation by Hf-DBP MOF and Hf-QP MOF (40 μM Hf) under X-ray irradiation. QP is amide-quaterphenyldicarboxylic acid, a ligand without photosensitizing properties. All data are shown as mean±SD, n=3.

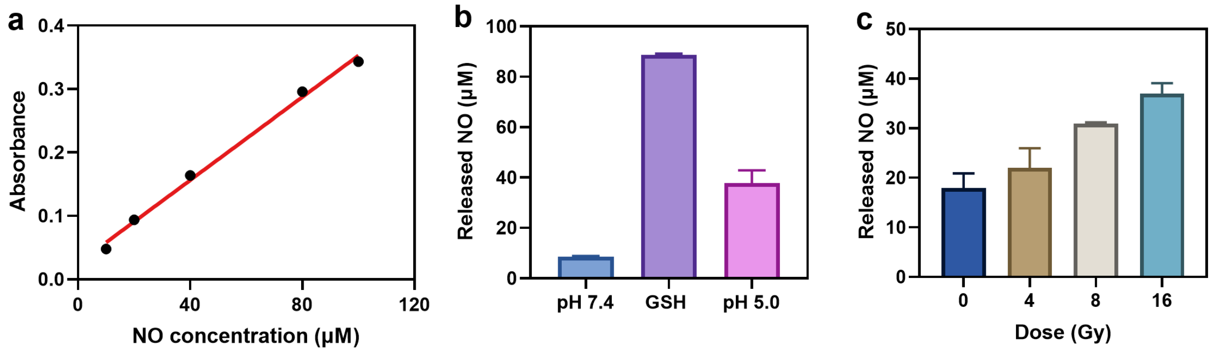

Figure S15. (a) NO standard curve obtained by Griess reagent test. (b) NO release from SNAP in different microenvironments. n=3. (c) NO release from SNAP/MOL after exposure to different doses of X-ray. n=3, all data are shown as mean±SD.

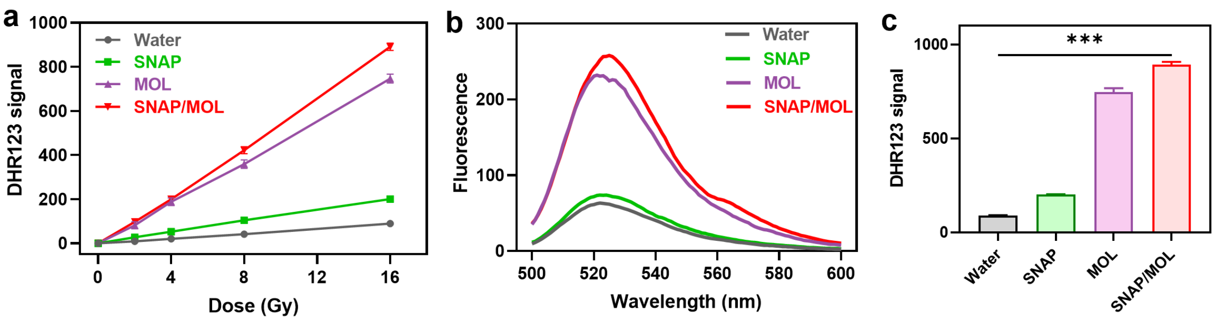

Figure S16. (a) DHR123 signals of SNAP, MOL, and SNAP/MOL at different doses of X-ray irradiation. (n=3) (b) Fluorescence spectra of DHR123 with different treatments. The Hf concentration was 40 μM, with 8 Gy X-ray irradiation. (c) DHR123 fluorescence intensities at 528 nm after 16 Gy X-ray irradiation. n=3, data are shown as mean±SD. SNAP-induced enhancement of DHR123 signal may be due to the generation of $ONOO^-$.

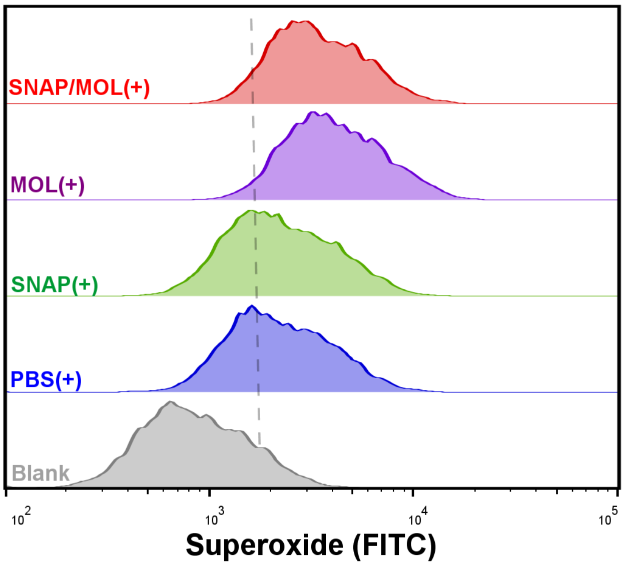

Figure S17. Fluorescence histograms showing superoxide generation in 4T1 cells under X-ray irradiation.

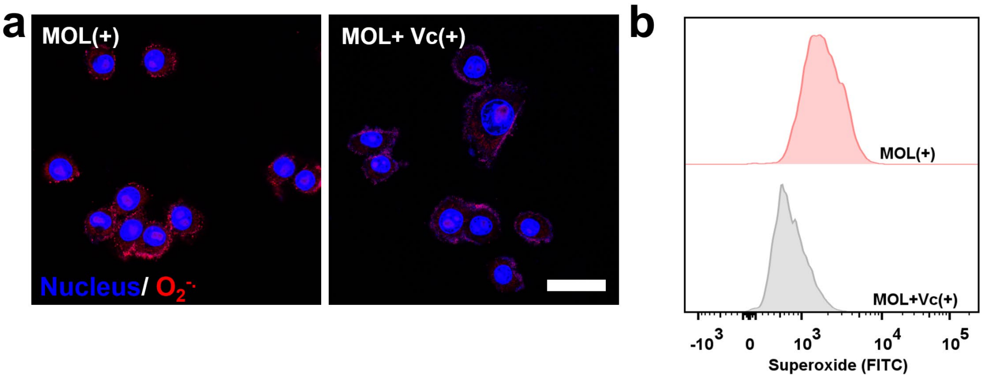

Figure S18. Confocal images (a) and flow cytometric analysis (b) showing the quenching of superoxide signals by Vc. Scale bar, 20 μm.

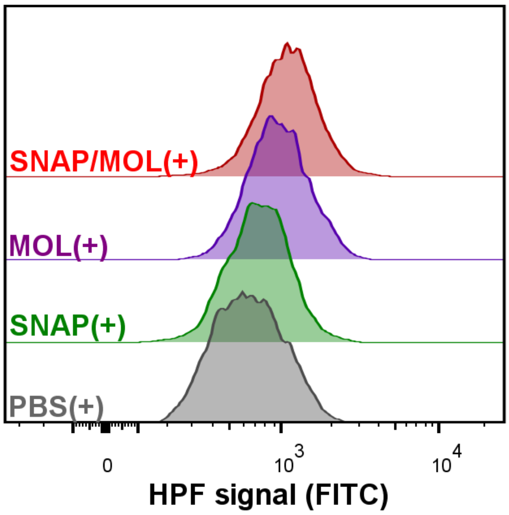

Figure S19. Fluorescence histograms showing hydroxyl radical generation under X-ray irradiation probed by HPF probe.

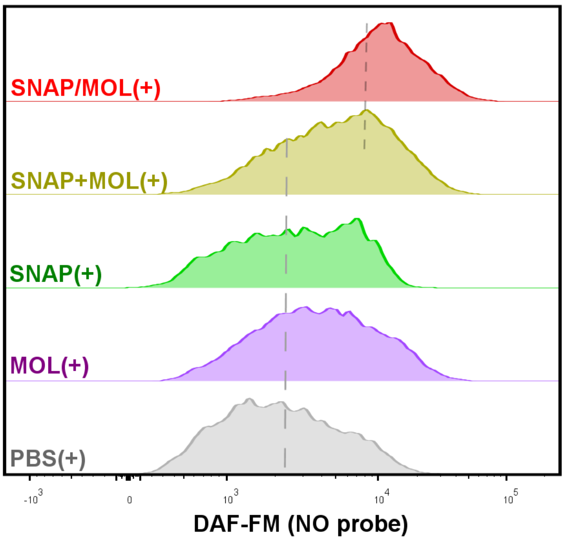

Figure S20. Fluorescence histograms showing NO generation in 4T1 cells under X-ray irradiation as probed by DAF-FA DA.

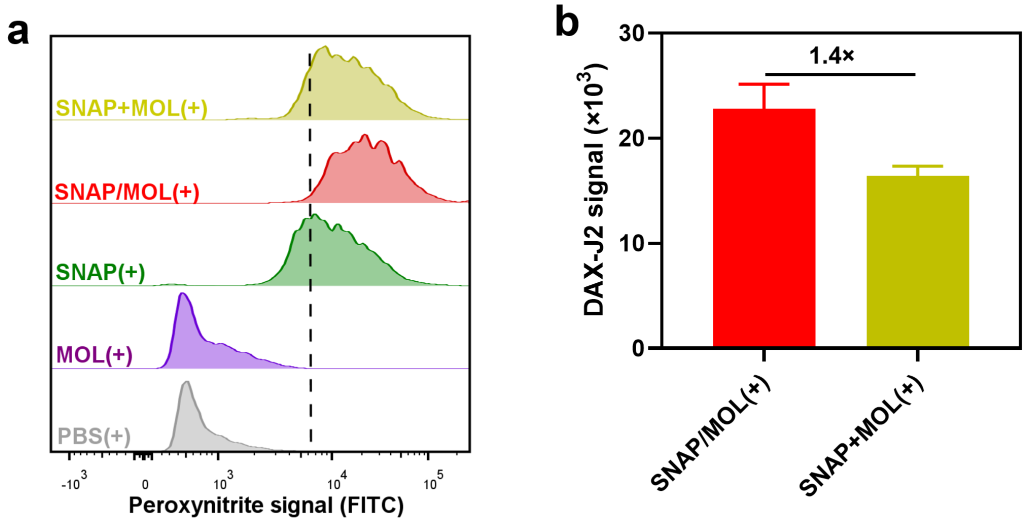

Figure S21. (a) Fluorescence histograms of intracellular peroxynitrite signals by flow cytometry using DAX-J2 probe. (b) Intracellular DAX-J2 signal intensity statistics after SNAP/MOL(+) and SNAP + MOL(+) treatments (n=3, data are shown as mean±SD).

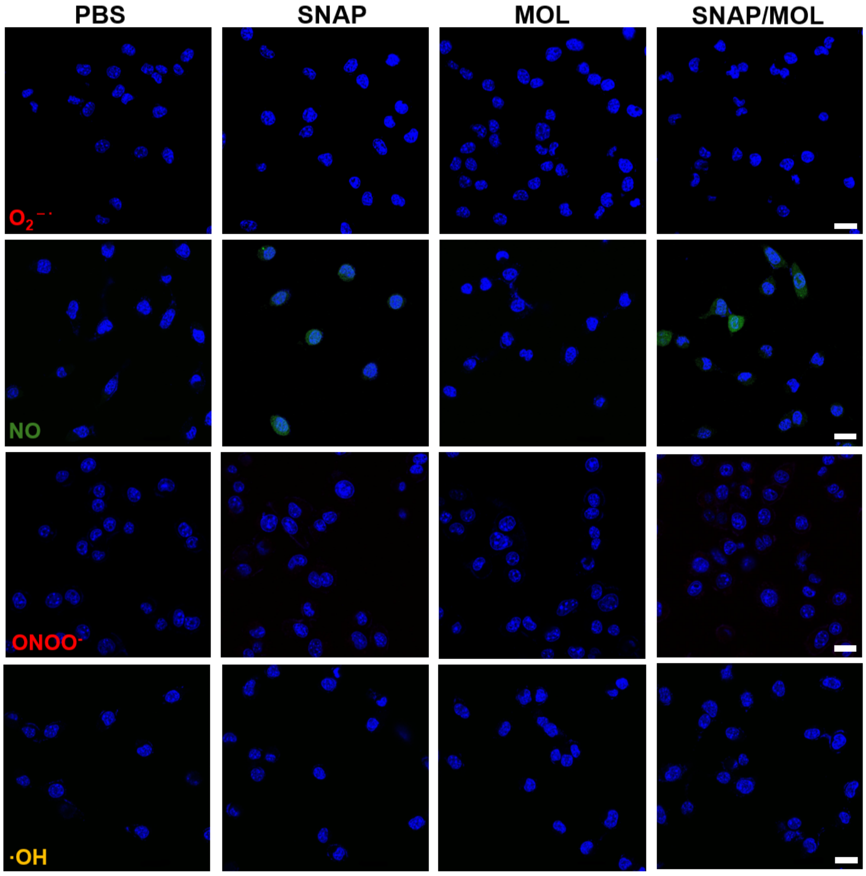

Figure S22. CLSM images of (a) $O_2^{-\cdot}$, (b) NO, (c) $ONOO^-$ and (d) ·OH staining of different groups without X-ray irradiation. The nuclei were visualized with Hoechst 33342 (blue). The scale bars are 20 μm.

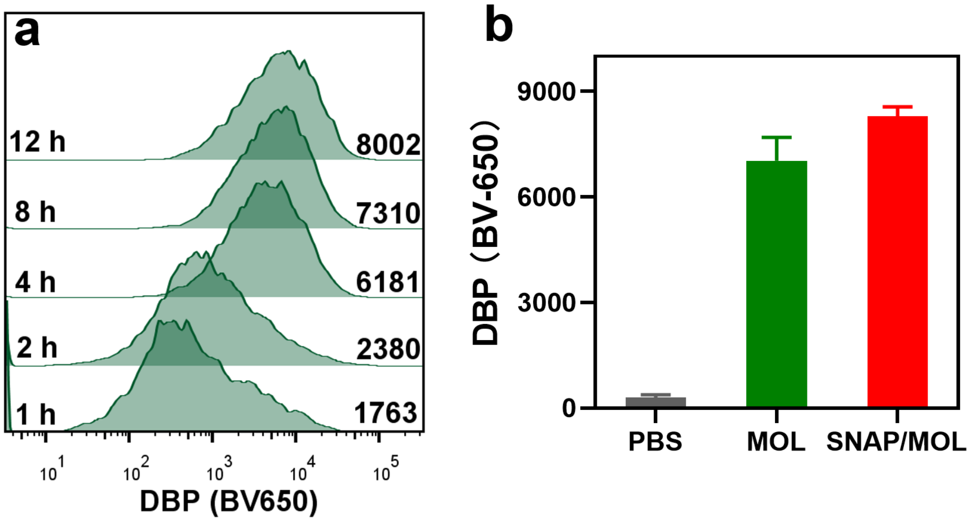

Figure S23. (a) Time-dependent uptake of SNAP/MOL in 4T1 cells via flow cytometric analysis of DBP fluorescence. (b) DBP fluorescence intensities after 12-hour incubation of 4T1 cells with MOL and SNAP/MOL by flow cytometry (n=3, data are shown as mean±SD).

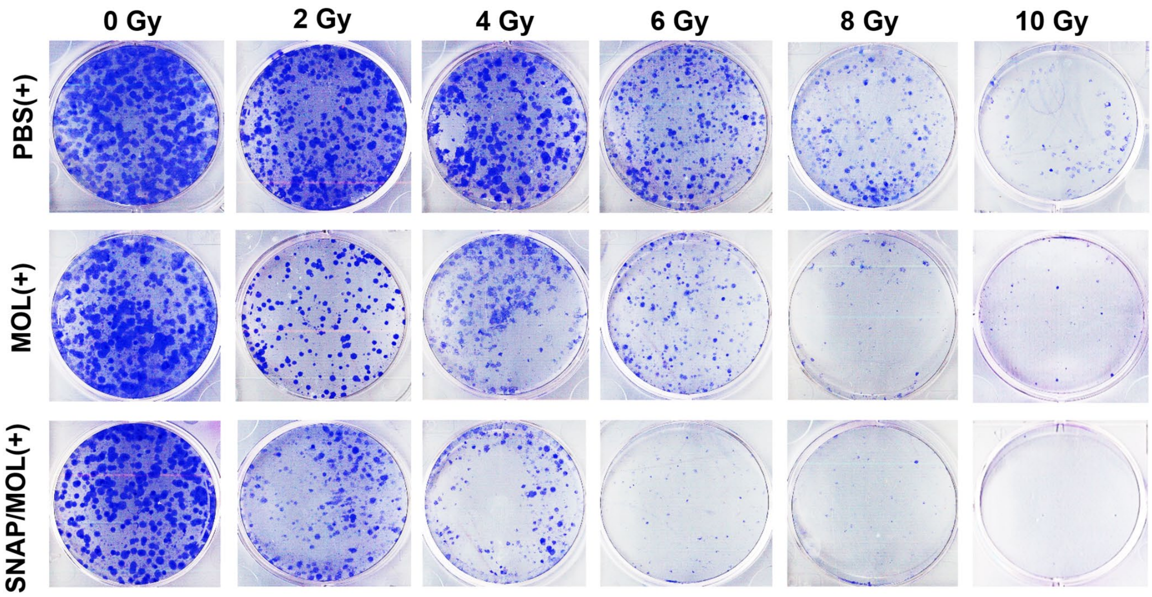

Figure S24. Representative images of colonies formed by 4T1 cells treated with PBS, MOL, or SNAP/MOL under different doses of X-ray irradiation.

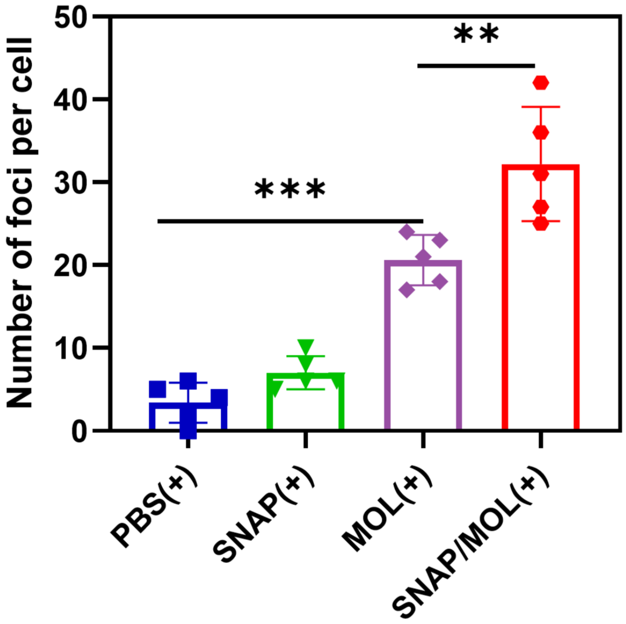

Figure S25. Statistical analysis of γ-H2AX assays showing DSBs in 4T1 cells after different treatments. All data are shown as mean ± SD, n=3. Statistical significance was calculated via unpaired two-tailed student's t test. ** p< 0.01, *** p < 0.001.

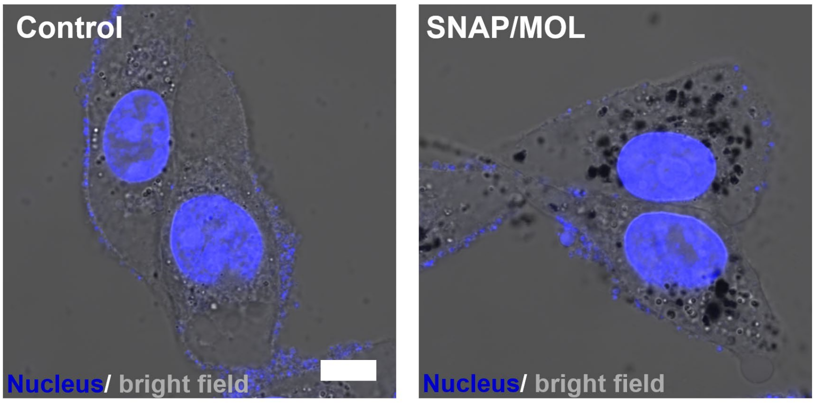

Figure S26. CLSM images showing the distribution of SNAP/MOL in 4T1 cells.

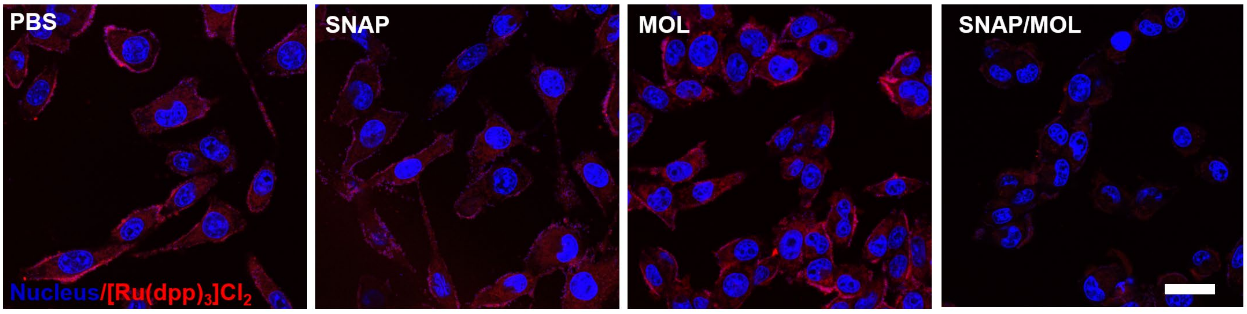

Figure S27. Confocal images of $Ru(dpp)_3Cl_2$ luminescence signals in 4T1 cells treated with SNAP, MOL and SNAP/MOL in a hypoxia chamber (pO_2 = 0.5).

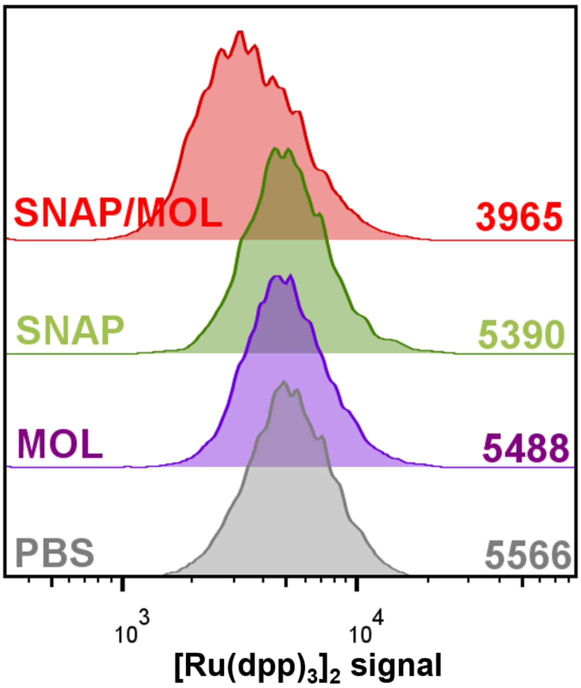

Figure S28. Histograms showing Ru(dpp)$_3$Cl$_2$ luminescence intensity in 4T1 cells with different treatments.

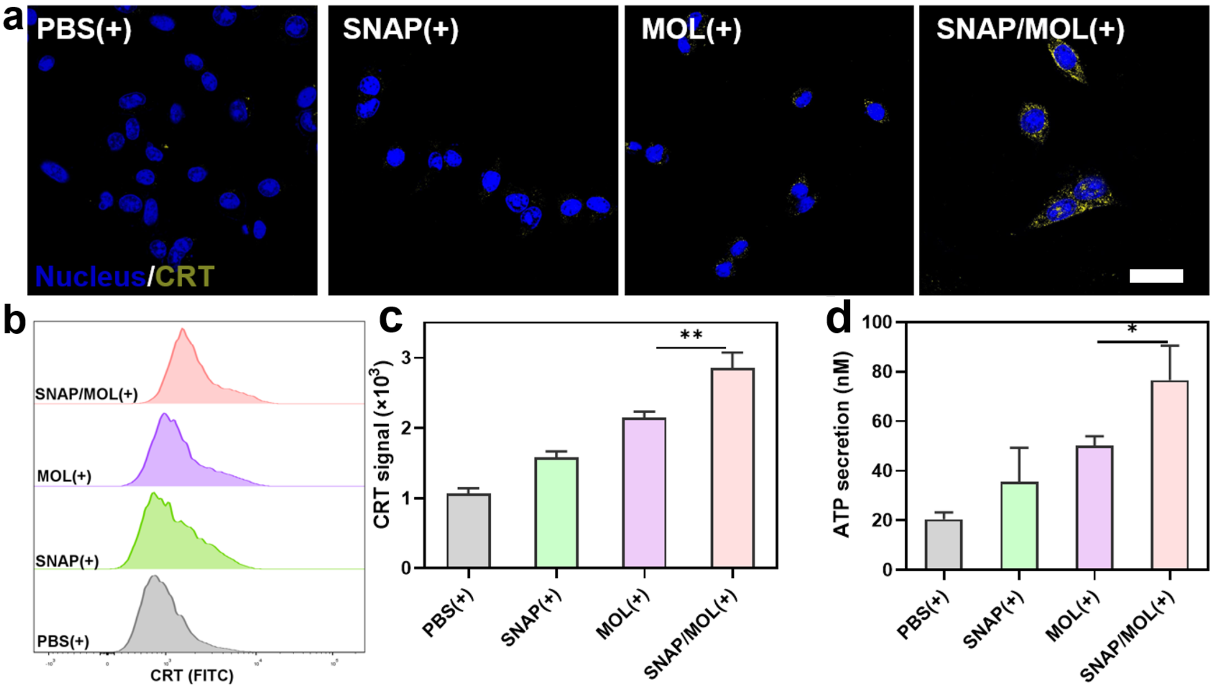

Figure S29. (a) CLSM images of surface translocation of CRT of 4T1 cells after different treatments. (b) Flow cytometric analysis of CRT expression on 4T1 cells 24 h after different treatments and (c) quantitative analysis of the corresponding CRT fluorescence intensities. (d) ATP secretion from 4T1 cells after indicated treatments measured by ATP Determination Kit. n=3 for each experiment. All data are shown as mean ± SD. Statistical significance was calculated via unpaired two-tailed student's t test. * $p<0.05$, ** $p< 0.01$.

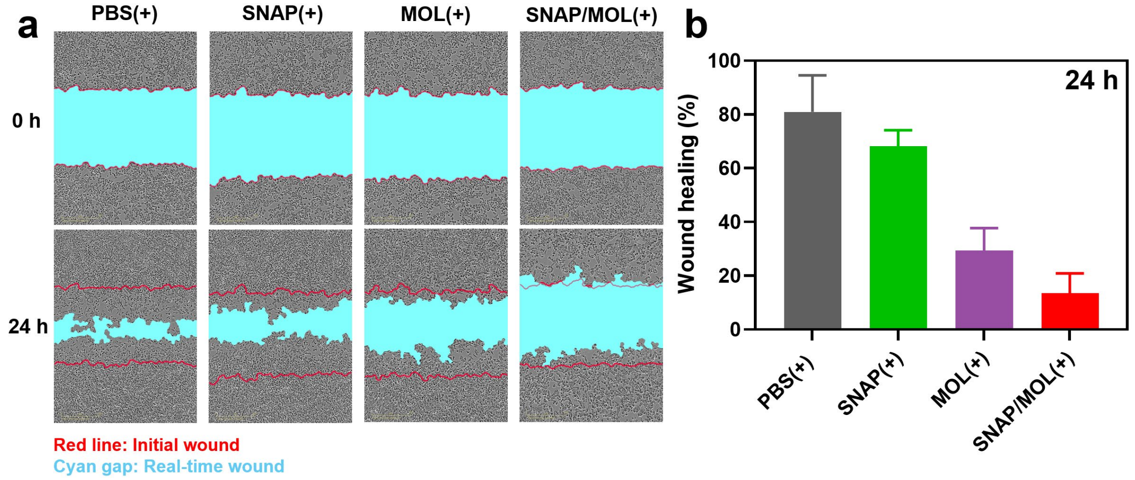

Figure S30. (a) Representative images of wound healing at 0 h and 24 h after radiation treatments. The initial wound is outlined in red, while the current wound is indicated by the cyan gap. (b) Statistical results of wound healing rate at 24 h (n=4). All data are shown as mean ±SD.

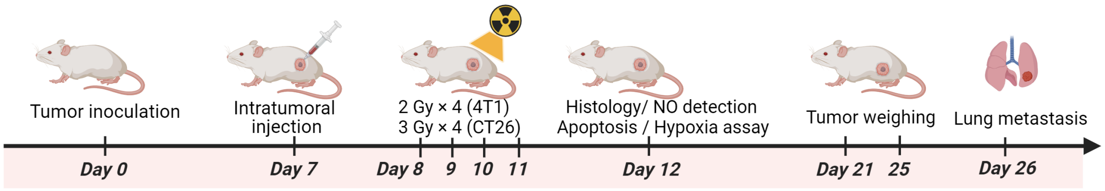

Figure S31. Experimental protocols for anti-tumor studies.

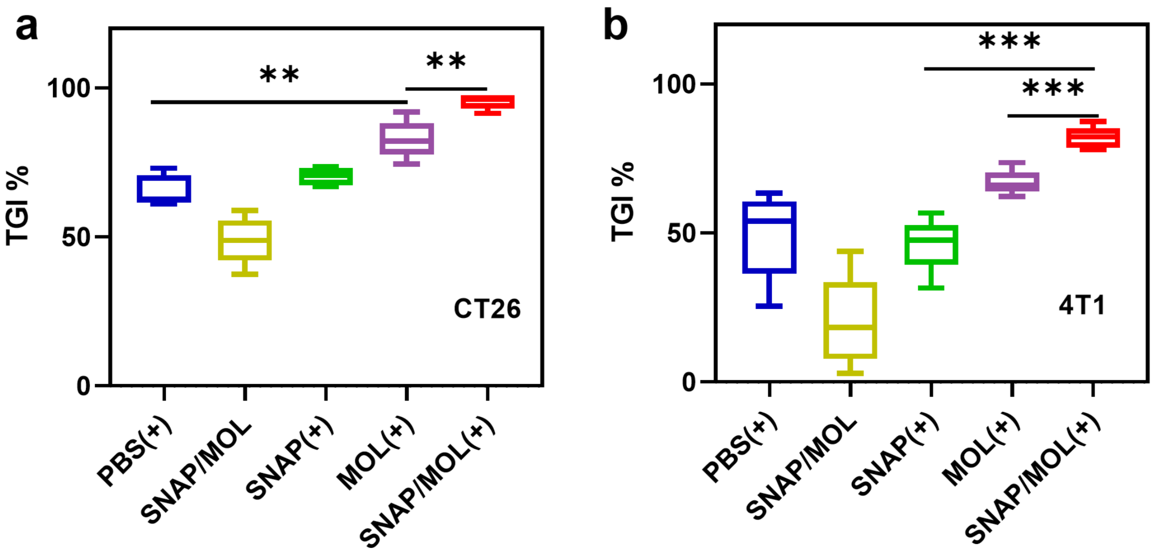

Figure S32. (a) Tumor growth inhibition (TGI) values of CT26 model calculated from tumor volume data on day 19. (b) TGI values of 4T1 model calculated from tumor volume data at the endpoint. n=5. All data are shown as mean±SD. Statistical significance was calculated via unpaired two-tailed student's t test. ** $p< 0.01$, *** $p < 0.001$.

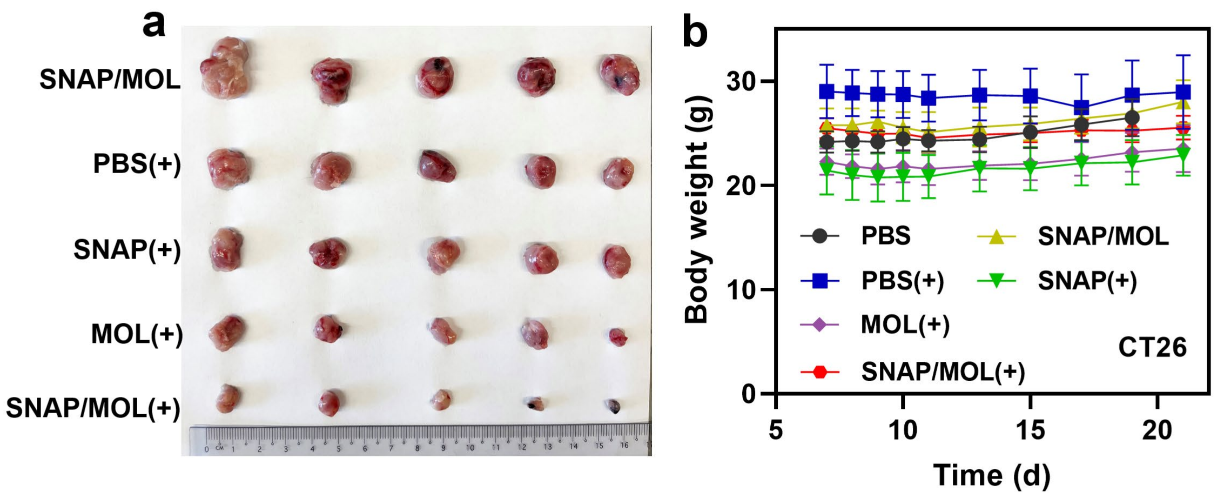

Figure S33. (a) *Ex vivo* images of CT26 tumors extracted from mice at the end of treatments. (b) Body weights of CT26 tumor-bearing mice during the treatment period. n=5. All data are shown as mean±SD.

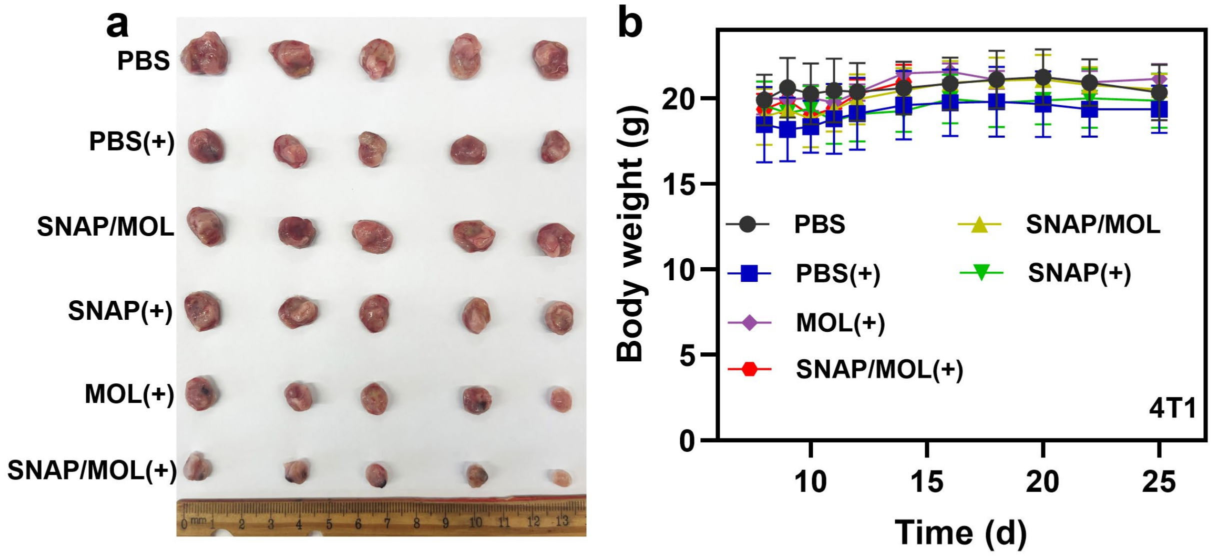

Figure S34. (a) *Ex vivo* images of 4T1 tumors extracted from mice at the end of treatments. (b) Body weights of 4T1 tumor-bearing mice during the treatment period. n=5. All data are shown as mean±SD.

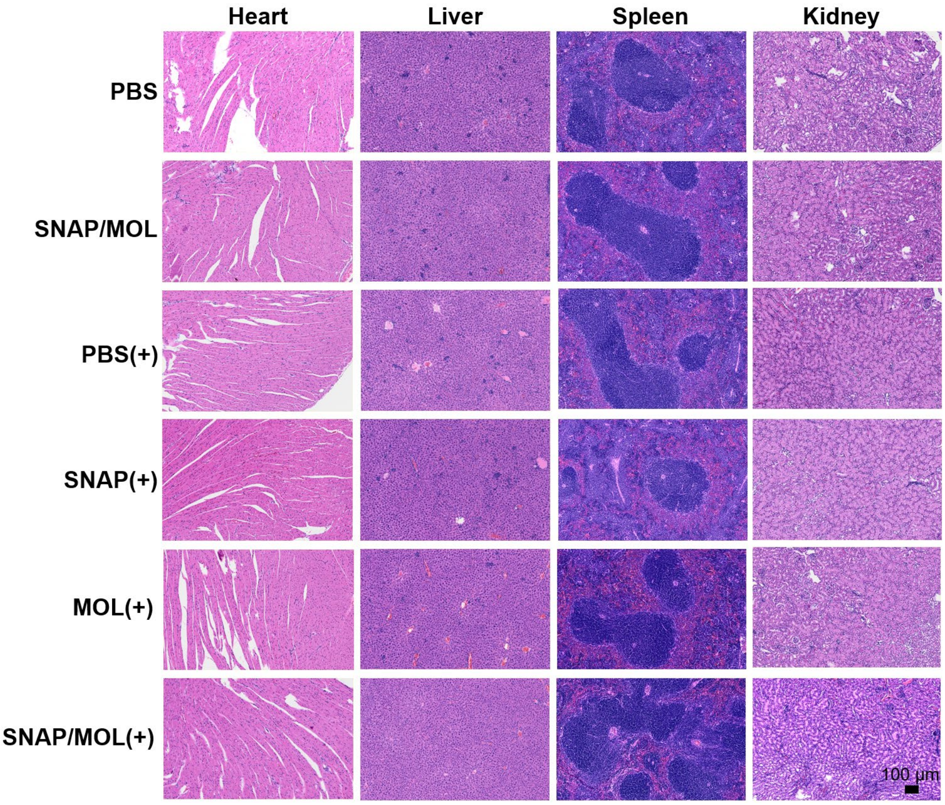

Figure S35. H&E sections of major organs of 4T1-bearing BALB/c mice.

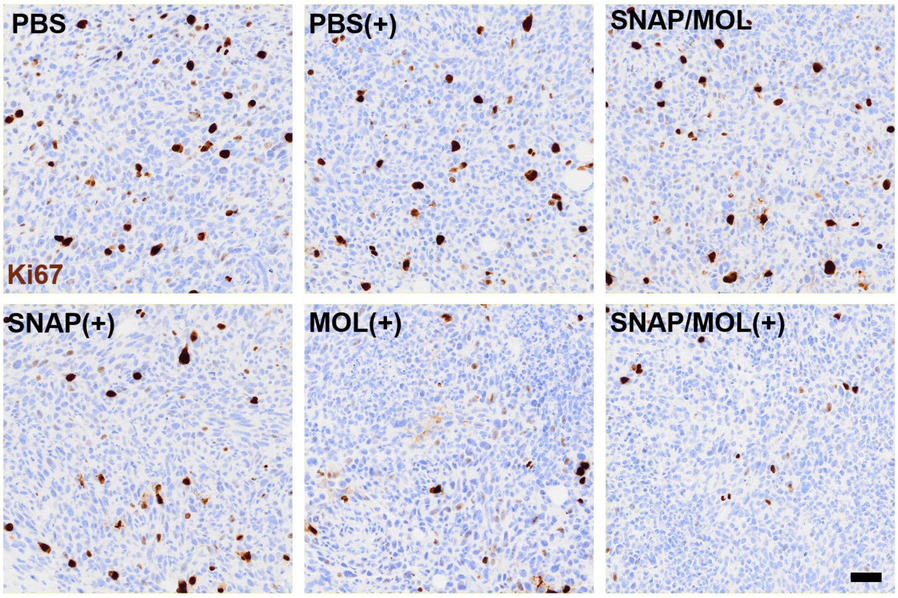

Figure S36. Immunohistochemistry of Ki67 staining of excised 4T1 tumors. Scale bars, 50 μm.

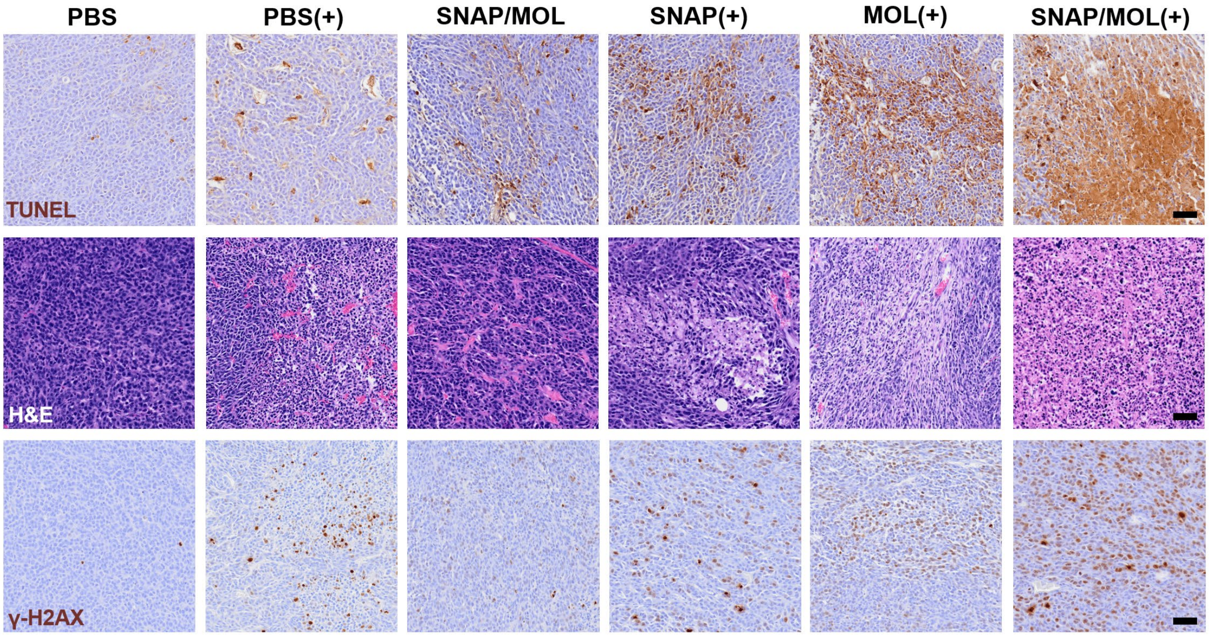

Figure S37. TUNEL, H&E and γ-H2AX staining of CT26 tumors resected after different treatments. Scale bars, 50 μm. The results showed that SNAP/MOL(+) treatment induced intense apoptosis, severe necrosis, higher DNA damage on CT26 tumor modal.

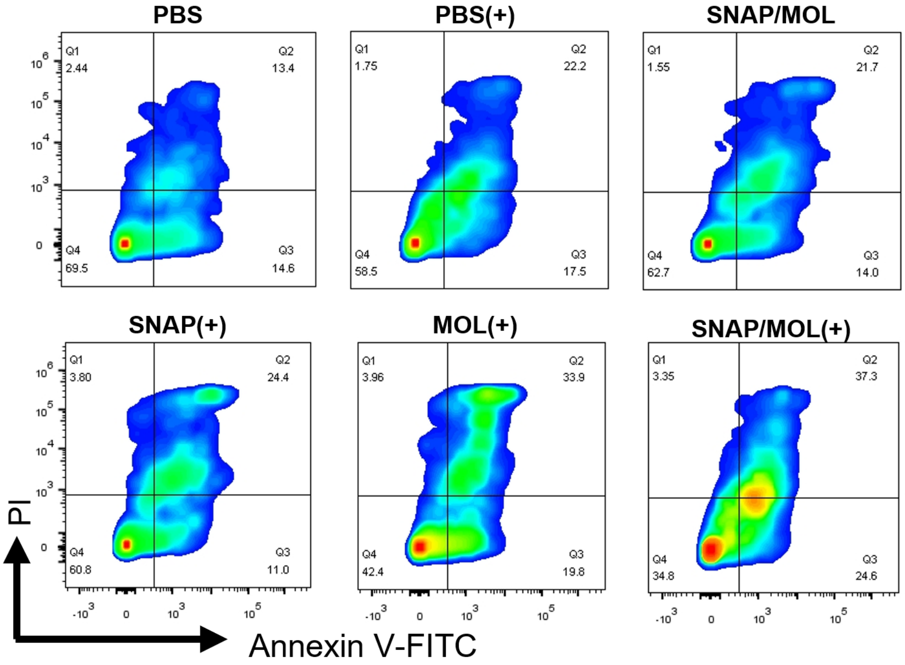

Figure S38. Assessment of apoptosis in 4T1 tumor tissues after different treatments.

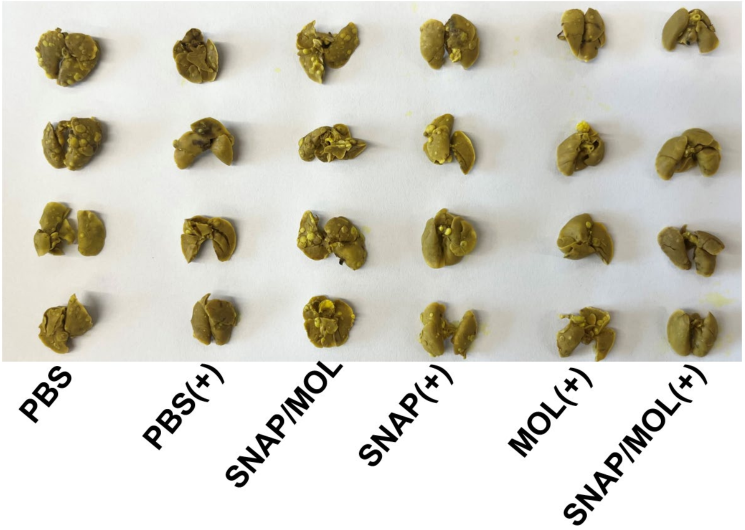

Figure S39. Photographs of the gross appearance of the tumor nodules in the lung after different treatments.

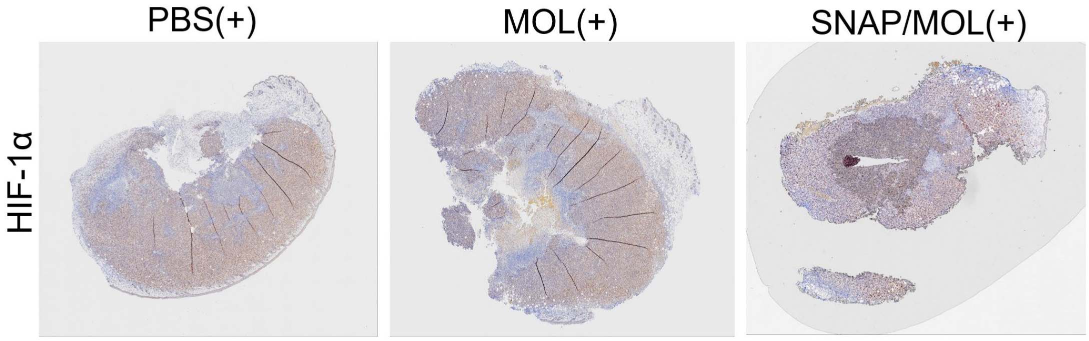

Figure S40. Immunohistochemistry analysis of HIF-1α staining in whole tumors.
